# Research on the Stability Model in Discrete Dynamical Systems with the Lorenz Attractor and the Kropotov–Pakhomov Neural Network

**DOI:** 10.3390/e28010012

**Published:** 2025-12-22

**Authors:** Ekaterina Antonova Gospodinova

**Affiliations:** Technical University of Sofia, Sofia 1000, Bulgaria; ekaterina_gospodinova@tu-sofia.bg

**Keywords:** stability theory and strategies, discrete dynamical systems, Lorenz attractor, Kropotov–Pahomov neural network, chaotic systems

## Abstract

This paper explores the dynamic analogy between the discrete Lorenzian attractor and a modified Kropotov–Pakhomov neural network (MRNN). A one-dimensional peak map is used to extract the successive maxima of the Lorenzian system and preserve the basic properties of the chaotic flow. The MRNN, governed by the Bogdanov–Hebb learning rule with dissipative feedback, is formulated as a discrete nonlinear operator whose parameters can reproduce the same hierarchy of modes as the peak map. It is theoretically shown that the map multiplier and the spectral radius of the monodromy matrix of the MRNN provide equivalent stability conditions. Numerical diagrams confirm the correspondence between the control parameters of the Lorenz model and the network parameters. The results establish the MRNN as a neural emulator of the Lorenz attractor and offer an analysis of self-organization and stability in adaptive neural systems.

## 1. Introduction

The study aligns with the growing interest in discrete dynamical systems, where discretization serves not only as a numerical tool but also gives rise to new structural and dynamical properties. Despite significant progress in analyzing discrete Lorenz attractors and neural architectures, there is no unified framework that connects the stability of chaotic maps to adaptive neural models. The present work fills this gap by combining the discrete Lorenz attractor and a modified Kropotov–Pahomov neural network (MRNN). The paper presents a discretization of the Lorenz system and a one-dimensional peak map that maintains the fundamental bifurcation and chaotic properties. The MRNN is formulated as a discrete nonlinear operator trained using the Bogdanov–Hebb rule. In parallel with the emergence of new applied fields of science, where the study of discrete mathematical–algorithmic constructions is the basis of the research methodology, there is a clear tendency to rethink the adequacy of the description of physical reality from the perspective of constant mathematics [1,2,3,4], which is not in the least due to the intensive research of chaotic systems.

Discrete dynamical systems are a natural environment for studying stability, bifurcations, and chaos, as they combine the analytical traceability of local features, such as the Jacobi spectrum [5,6,7,8], with rich global behavior. Of particular interest are discrete Lorenz attractors, which reproduce key features of the classical Lorenz attractor but arise in three-dimensional representations with different symmetries and a constant Jacobi [9,10,11]. Recently, a wide class of 3D maps has been shown to admit discrete Lorenz attractors, and their bifurcation picture has been systematized, which provides a solid basis for quantitative analysis of stability in discrete time. The classical continuous Lorenz system [12,13,14] remains a canonical example of the transition from stationary solutions to chaos. The practical stability limits and estimates of the attractor dimensionality are still the subject of active research today. Discrete analogies that explore the conditions for loss of stability and the role of local/global invariants motivate these results. In parallel, work is developing in the field of neuroscience and computer modeling on “realistic” neural networks of the Kropotov–Pakhomov type, which aim to capture the dynamics of cortical populations through simple but biologically motivated nonlinearities and local interactions [15,16,17,18]. The original publications from the 1980s laid the foundations of the model, taking into account the influence of synaptic plasticity. Later studies modified it and analyzed it in terms of stability and structure formation. Neural models can approximate dynamics and derive local stability properties, such as the Jacobian–Lyapunov spectrum, directly from observations [19,20,21]. In [22,23,24,25,26], researchers propose methods for extracting Jacobians and stability indices from data using recurrent architectures that also allow stability analysis for discrete Lorenz maps [27,28,29,30,31,32]. Studies of discrete dynamical systems with chaotic behavior have shown that even a simple system of three differential equations can exhibit stable chaos. The analysis of Sparrow [13] and Shilnikov [14] formulated the criteria for the emergence of so-called Lorenz attractors and extended the concept to discrete images. In recent decades, refs. [33,34,35,36,37] systematized the classes of discrete Lorenz attractors, proving their structural stability and characteristic spiral behavior in the space of phase variables.

Regarding the stability of discrete chaotic systems, several studies have used local spectral analysis methods and Lyapunov exponents to estimate the boundaries between stable and chaotic regimes. The works of Ott, Grebogi, and York [38,39,40] initiated the concept of chaos control by small feedback, which is directly related to the stabilization of discrete Lorenz images. More recent studies by Chen, Zhang, and Wang [41,42,43] have applied neural models and machine learning to identify stable subspaces and predict bifurcations. The Kropotov–Paкhomov neural network was proposed in the late 1980s as a model of cortical ensembles with nonlinear synaptic integration and adaptive threshold excitation. The network is characterized by local recurrent connections and the ability to self-organize in stable states, making it suitable for studying stability and transitions between quasi-stationary regimes. Later modifications by Kropotov–Paкhomov and Shaposhnikova [44] integrate elements of dynamical systems theory and allow its application in cognitive modeling and signal processing. Combining discrete chaotic systems with neural networks is a current trend in modern nonlinear sciences. Using neural predictors to reconstruct attractors (e.g., via autoencoder or recurrent network), the approaches described in [19,20] are standard methods. Trainable models, as demonstrated in [45,46,47], can extract the internal dynamical invariants of complex systems from time series alone. The result opens up possibilities for data-driven stability analysis, as explored in the present work, to combine the discrete Lorenz attractor with the Kropotov–Pakhomov neural architecture as a tool for stability assessment and prediction.

The mathematical understanding contains elements that are partially irreducible to algorithmic methods [48]. Despite the presence of mutually exclusive positions in the approaches described to the study of chaotic systems, none of them can be completely abandoned. Control over a chaotic dynamical system means a change or external influence that excludes its chaotic behavior. It is crucial to observe the minimal nature of the corresponding changes or influences. For the Lorenz system, the introduction of a map whose nodes do not coincide with the origin of coordinates, which is a stable fixed point, practically eliminates the dependence of the control result on the initial conditions, since almost all trajectories fall into one cycle of the attractor. The ability to change the parameters without changing the structure of the attractor allows the method to be applied to real physical systems to regulate their chaotic dynamics. Due to the stochastic dependence of the attractor’s structure on the grid step and the accuracy scale, near each point of the attractor, there are not only continuous intervals where the attractor is represented by a simple cycle, but also sections where the attractor exhibits a predetermined structure based on the permissible upper limit of fluctuations, which is determined by the current accuracy. Therefore, each accuracy value can be associated with two characteristic scales: an error and an interval, which defines the neighborhood around a point where the existence of a corresponding attractor structure is guaranteed. For a fixed accuracy, the discretized system is regular. Its dynamics are stable with respect to variations that are smaller than the error. If the discretization process fails to maintain the map step within certain limits, the outcome leads to an irregular discrete system. The stability and dynamics of complex nonlinear systems are modeled by combining the classical theory of dynamical systems with modern methods of neural learning.

The main shortcomings that continue to pose scientific challenges include the following:

1. Lack of a universal stability metric—most methods measure stability through local indicators, but there is no global criterion compatible with both discrete and stochastic systems.
2. Sensitivity to parameters and initial conditions—chaos control and neural predictions often destabilize with minimal changes in parameters, and training becomes unstable.
3. Low interpretability of neural models—autoencoders and reservoir networks extract dynamic invariants, but it is not clear how they correspond to the mathematical model (Lorenz et al.).
4. Limited portability between systems—a trained model (e.g., for Lorenz) is a challenge to adapt to a similar but different chaotic regime.
5. Incomplete unification of approaches—dynamical systems theory is analytical and deterministic; neural methods are stochastic and approximate. There is still no strict mathematical connection between them.

The Kropotov–Pakhomov neural model integrates elements of dynamical systems theory in the context of cognitive modeling and signal processing. Machine learning approaches, especially those using autoencoders and recurrent networks, show similar trends.

Despite these achievements, several significant limitations remain. First, there is no universal metric for assessing stability. Applied learning is sensitive to parametric variations and often suffers from low interpretability with respect to real dynamic variables. Third, the relationship between theoretical chaos models and empirical neural representations has not yet been formalized.

### Dynamic Mathematical Concepts Used in the Article

Fundamental concepts from the theory of dynamical systems, discrete iterations, and neural dynamics underlie the analysis presented in the article:

1. Nonlinear dynamical systems and their phase space. Systems are described by nonlinear equations in continuous or discrete time:
  - Continuous systems: $\dot{X}$ = *F*(*X*);
  - Discrete iterations: $X_{k+1}=G(X_{k})$.

The phase space represents the set of all possible states.

An invariant set is a subset of the phase space that is closed under the dynamics. An attractor is a compact invariant set that attracts neighboring trajectories. The paper uses the continuous Lorenz attractor, the discrete Lorenz attractor, and the MRNN neural attractor. The paper examines how the bifurcation structures of the peak map *P* in MRNN dynamics change when η and γ vary:

2. The reduction in a continuous Lorentz system to a one-dimensional peak map, and P:I⟶I is a standard method in dynamical systems: $x_{n+1}$= *P*($x_{n}$). This captures bifurcations, periodicity, and chaos through discrete reduction.
3. The spectrum of the Jacobian determines the stability of a fixed point or periodic orbit in discrete time: $X_{k+1}=G\left(X_{k}\right),\;\;J=DG\left(x^{*}\right).$ The criterion is ρ(J)⇔ asymptotic stability. In the article, this criterion is associated with the derivative. $P^{\prime }{(u}^{*})$ for a 1D map and the spectral radius $\rho (M_{T})$ of the MRNN.
4. The monodromic matrix describes the behavior of variations along a T-periodic orbit: $M_{T}=\prod_{j-0}^{T-1}DF(X_{j})$, where stability is determined by $\rho {(M}_{T})<1.$ The matrix is involved in Theorem 1.
5. An orbit or attractor is hyperbolic if all eigenvalues satisfy stable directions: |λ|<1, unstable: |λ|>1. In Theorem 1, the tangent direction along a 1D invariant manifold corresponds to transverse eigenvalues $\left|\lambda_{trans}\right|>1$, which guarantees a reduction to 1D.
6. Diffeomorphisms and dynamic conjugacy: two systems *P* and are *F* dynamically conjugate if there exists a differentiable transformation: H∘F=P∘H. This means that the systems have the same dynamics up to a change in coordinates. It is used to connect MRNN and a 1D map.
7. The 1D invariant manifold (*M*) in MRNN allows for reduction ${F|}_{M}\sim P.$ This is the structural basis of Theorem 1.
8. Recurrent dynamic operators and neural maps. MRNN is represented as a discrete operator: $X_{k+1}=F\left(X_{k},\eta ,\;\gamma \right).$ This allows for classical stability analysis via spectral radius, local derivatives, and invariant subspaces.
9. An important role is played by the theory of dimensionality reduction and invariant manifolds. If there exists a *C*^1^ embedded one-dimensional manifold *M* ⊂ $R^{m}$, continuously invariant for MRNN, and a diffeomorphism *H*: *M* → *I*, such that $H\circ F_{m}=P\circ H$. Then, MRNN and the Lorenz peak map are conjugated on this manifold. Conjugation guarantees correspondence between local stability indices:

$\rho \left(M_{T}\right)=|\prod_{k-0}^{T-1}P^{\prime }(z_{k})$. This provides a rigorous mathematical basis for deriving the Stability Correspondence Theorem, which is the main analytical contribution of the paper.

The work uses concepts from chaos theory in networks: dissipativity, nonlinear feedback, normalizing operators, and Hebbian-type adaptation in time. These mechanisms guarantee the existence of compact invariant sets for MRNN and allow a comparison of its dynamics with the reduced Lorenz map. Thus, the mathematical framework connects Jacobian analysis, Lyapunov indicators, dynamic reduction, and bifurcation structures into a unified system for comparison and proving equivalence.

The present study proves equivalence between the peak map multiplier and the spectral radius of the monodromy matrix of MRNN as a general stability criterion. Numerical experiments demonstrate a functional analogy between the two systems and the ability of MRNN to reproduce stable, bifurcated, and chaotic regimes. The results offer a conceptual bridge between chaos theory and learning neural systems and provide a new tool for analyzing and predicting stability in discrete nonlinear processes. The main goal is to develop a model for evaluating and analyzing stability in discrete dynamical systems, based on the integration between the discrete Lorenz attractor and the Kropotov–Pakhomov neural network. This model combines classical methods for chaos analysis with learning architectures capable of extracting and adapting internal system parameters based on observed time series.

The expected contribution of the study is conceptual and applied. From a theoretical perspective, it proposes a new approach for integrating discrete chaotic models and neural systems, allowing stability to be described not only by classical metrics but also by dynamical characteristics extracted from trained architectures. From a practical perspective, the model provides an adaptive tool for predicting and controlling discrete nonlinear processes, applicable in areas such as cognitive modeling, signal processing, and systems with chaotic responses.

The rest of this paper is organized as follows: Section 2 introduces several topics, including the discretized Lorenz system, invariant sets and attractor structures, the fourth-order Runge–Kutta (RK4) method, various discretization methods, a Lorenz time discretization algorithm applicable to arbitrary systems, and a linear stability criterion. Section 3 presents the modified Kropotov–Pakhomov neural network model. This section discusses the original and modified models, the dynamic regimes in a realistic model, the Bogdanov–Hebb learning rule, the stability criteria of the modified model, and the entropy of desynchronization and connections. This section presents some examples. Section 4 is a discussion and results section, where the correspondence between the discrete Lorenz attractor and the modified Kropotov–Pakhomov neural network (MRNN) is derived. By introducing the vertex map, the Lorenz system is reduced to a one-dimensional discrete representation that preserves its fundamental bifurcation and chaotic properties, defining the stability criteria in both systems. The functional analogy between the continuous chaotic dynamics of the Lorenz system and the adaptive self-organization of the MRNN is illustrated with graphs and diagrams. Section 5 concludes the study.

## 2. The Discrete Lorenz System

The classical Lorenz system is described by a system of three ordinary differential equations of the following form:

(1) $$\{\begin{array}{c} \dot{x}=\sigma \left(y-x\right)\\ \dot{y}=x\left(\rho -z\right)-y,\\ \dot{z}=xy-\beta z \end{array}$$

where the parameters σ, ρ, and β > 0 determine the nature of the motion and the possibility of a chaotic attractor. An invariant and singular-hyperbolic organization characterizes the global structure of the attractor. The attractor is a compact invariant set *A* ⊂ $R^{3}$, generated by iterations of a nonlinear operator T. There is a dominated bundle, *T* × $R^{3}$ = *E^s^*(*X*) ⊕ *E^cu^*(*X*), with exponential contraction in *E^s^* and volume expansion in *E^cu^* (pseudohyperbolicity). This guarantees robust chaoticity and structural stability under small parameter perturbations.

Unstable periodic orbits (UPO) are the basis. A is the closed hull of the UPO. Each UPO intersects Σ at a finite number of points. It is encoded by a finite set ${\left\{L,\;R\right\}}^{*}$. Their union sets the topological template of the attractor and controls the global transitions between the lobes. The geometry manipulators are either stable or unstable manifolds. The stable manifold $W^{s}$ forms a lamination that “folds” the trajectories back to A, while the unstable $W^{u}$ generates a bifurcation in both lobes. The interlacing around saddle equilibria determines the global chaotic structure [49]. The discretization of the system can be performed using several approaches: the Euler method; the Runge–Kutta method, which offers higher accuracy in approximating derivatives and better preservation of the structural properties of the attractor; implicit and hybrid methods; and nonlinear and adaptive schemes.

In this section, the term “space-time discretization” does not mean discretization of space in the sense of partial differential equations. The Lorenz system is an ODE that has only one independent variable, time. The term used refers to a standard approach in numerical theory, in which discretization in time is combined with state evaluation at intermediate points in the phase space. Specifically, “space-time” discretization here means each time step $t_{n}\to t_{n+1}$ uses intermediate values $x_{n+\frac{1}{2}},\;y,\;z_{n+\frac{1}{2}}.$ These represent centroids, or mean estimates, in the state space, rather than in a physical geometric space. This allows for a more accurate numerical approximation of the nonlinear terms, reduces local error, and preserves certain structural properties of the original continuous system.

Therefore, the discretization under consideration is entirely time-based, and the “spatial” component refers only to the phase coordinates of the system, not to the PDE context.

The Lorenz system is a differential equation that does not have spatial coordinates in the sense of a PDE. In the method under consideration, only time is discretized $t_{n}=t_{0}+nh$, but a rectangular approximation of the nonlinear terms uses average values in the space of phase variables, also called state-space discretization. The discretization is given by an operator $\Phi_{h}:$ $x_{n}\to x_{n+1}$, constructed as follows:

(2) $$x_{n+1}=x_{n}+hf(\frac{x_{n}+x_{n+1}}{2}).$$

This is a modified centroid point. Here, $\frac{x_{n}+x_{n+1}}{2}$ is not a spatial coordinate but an average position of the trajectory in the three-dimensional state space.

Therefore, “spatial” refers to the state space $R^{3}$ and not to a physical space.

The scheme involves discretization in time as follows:

$$t_{n}\to t_{n+1}=t_{n}+h.$$

Evaluating the nonlinear terms at an intermediate point in the phase space is nontrivial because f(x,y,z) is nonlinear.

This dual aspect (time step + phase averaging) is called spatio-temporal discretization in the nonlinear ODE literature, although “spatio” means state space.

The equivalent formulation, expressed as an update function, is the implicit scheme that results in a discrete map:

(3) $$x_{n+1}=\;G_{h}(x_{n}),$$

where $G_{h}$ is implicitly defined by

(4) $$x_{n+1}-\frac{h}{2}f\left(x_{n+1}\right)=x_{n}-\frac{h}{2}f\left(x_{n}\right).$$

### 2.1. Invariant Sets and Attractor Structure

The attractor can be described as a set:

(5) $$Å=\{X_{k}\in R^{3}:X_{k+1}\}=T\left(X_{k}\right),$$

where X is the state vector of the Lorenz system and $X_{k}$ is bounded as k→∞.

The fractal dimension D_L_ can be estimated using the Kaplan–York formula:

(6) $$D_{L}=j+\frac{\sum_{i-1}^{j}\lambda_{i}}{\left|\lambda_{j+1}\right|},$$

where j is the largest index for which $\sum_{i-1}^{j}\lambda_{i}$ ≥ 0 and λ is a broadening constant.

In the context of neural modeling, each coordinate (x, y, z) can be viewed as a dynamically activated neural unit, and the system as a three-neuron recurrent network:

(7) $$u_{k+1}=W\phi \left(u_{k}\right)+b,$$

where ϕ(⋅) is a nonlinear activation, and W  and  b are trainable parameters that can be calibrated so that the network reproduces the dynamics of the Lorenz attractor [21,22].

### 2.2. Runge–Kutta Method (Fourth Order, RK4)

For higher accuracy and stability, the fourth-order Runge–Kutta method (RK4) is used:

(8) $$\{\begin{array}{c} k_{1}=F(X_{k})\\ k_{2}=F(X_{k}+\frac{h}{2}k_{1})\\ k_{3}=F\left(X_{k}+\frac{h}{2}k_{2}\right)\\ k_{4}=F\left(X_{k}+hk_{3}\right)\\ X_{k+1}=X_{k}+\frac{h}{6}\left(k_{1}+2k_{2}+2k_{3}+k_{4}\right), \end{array}$$

where h is the discretization step, which takes states and returns the derivative vector of the same dimension as X, and F is the vector field of the system, taking states and returning the derivative vector of the same dimension as X, and $k_{i}$, which are intermediate slopes (estimates of the derivative) that Runge–Kutta combines to obtain a more accurate next value. This makes the RK4 much more precise than Euler at the same h, so the vertex map and cyclic structures are preserved cleaner. RK4 is fourth-order accurate, with local error $O(h^{5})$ and global error $O(h^{4}).$ With a suitable choice of step $h\in [10^{-3},\;10^{-2}]$, the method preserves the phase structure and geometry of the Lorenz attractor while eliminating the unstable oscillations characteristic of the Euler scheme [50].

### 2.3. Discretization Methods

#### 2.3.1. Space Variables

In the analysis of nonlinear dynamical systems with chaotic nature—such as the Lorenz system—space variables are a generalization of classical numerical schemes, in which the temporal evolution process and the spatial dependencies between the states of the system are simultaneously discretized. This approach allows the modeling of distributed and interacting subsystems while preserving local stability properties and the topological structure of the attractor. In general, a continuous system can be represented by an operator form:

(9) $$\frac{\partial u(r,t)}{\partial t}=F\left[u\left(r,t\right)\right],$$

where u(r, t) is the state vector, r is the spatial coordinates, and F is the nonlinear operator describing the dynamics. The space-time discretization is implemented by dividing the continuous space into a finite number of cells (or nodes) and the time into a uniform or adaptive grid. The resulting system has the following form:

(10) $$u_{i}^{k+1}=u_{i}^{k}+hF_{h}\left(u_{i-1}^{k},\;u_{i}^{k},\;u_{i+1}^{k}\right),$$

where i is the spatial position index, k is the temporal layer, and h is the time step. Thus, the system is transformed into a network of discretely connected cells, each of which follows local Lorenzian oscillator-like dynamics. This approach allows one to model spatial correlations, wave structures of chaos, as well as local regions of stability and instability. In the context of neural systems, space variables are analogous to the introduction of a recurrent network with local connections, where each cell exchanges information with its neighbors [51,52].

#### 2.3.2. Centroid Space Variables

Centroid space variables are a generalization of the classical Euler and Runge–Kutta schemes [50], in which the evolution of the system is evaluated at a midpoint (centroid) between the current and the next state, both in space and time. This method provides better stability and symmetry in the approximation, preserving the integral invariants and reducing the numerical artifacts characteristic of chaotic maps. In general, the discretization for a system of the following form:

(11) $$\frac{\partial u}{\partial t}=F(u)$$

is set as the following:

(12) $$u_{k+1}=u_{k}+hF(\frac{u_{k}+u_{k+1}}{2}),$$

where $\frac{u_{k}+u_{k+1}}{2}$ is the centroid in time. In a spatio-temporal representation, each cell of the spatial grid is updated based on a local centroid calculated relative to its neighboring nodes:

(13) $$u_{i}^{k+1}=u_{i}^{k}+\;hF_{h}\left(\frac{u_{i-1}^{k},\;u_{i}^{k},\;u_{i+1}^{k}}{3},\;\frac{t_{k}+t_{k+1}}{2}\right).$$

This achieves an equilibrium estimate between spatial and temporal flows, minimizing local deviations and improving the robustness of the chaotic attractor in simulation [52]. An advantage of this approach is the possibility of using an adaptive centroid operator that automatically adjusts the spatial step according to the local sensitivity of the trajectory—a key property in the study of Lorenzian and Lorenz-like systems with variable robustness [53].

#### 2.3.3. Discrete Lorenz Attractor

The discrete Lorenz attractor is a numerical analog of the classical continuous attractor, obtained by discretizing the system of Lorenz equations on an appropriate time scale. With a proper choice of the discretization step h and parameters σ, ρ, and β, the system preserves the topological characteristics of the original chaos—sensitivity to initial conditions, attractors, and fractal structures—but also possesses new dynamical properties characteristic of iteration maps [54]. In the general case, the discrete Lorenz model is given by a system of equations:

(14) $$\{\begin{array}{c} x_{k+1}=x_{k}+h\sigma \left(y_{k}-x_{k}\right)\\ y_{k+1}=y_{k}+hx_{k}\left(\rho -z_{k}\right)-y_{k}\\ z_{k+1}=z_{k}+h(x_{k}y_{k}-\beta z_{k}) \end{array},$$

where h is the discretization step, and $\left(x_{k},\;y_{k},\;z_{k}\right)$ are the states of the system at time k. For small values of h, this system reproduces the behavior of the classical attractor. For larger values, new discrete chaotic regimes arise, as described in [50,55,56]. In the generalized discrete Lorenz maps, for which the nonlinear terms are modified with additional damping or noise functions, a stable simulation is achieved for larger values of h. Such maps were studied in [30,52], where the preservation of the characteristic “butterfly” structure of the attractor in the space (*x*, *y*, *z*) is demonstrated. The discrete Lorenz attractor can also be considered an iterative operator in the following phase space:

(15) $$T:R^{3}\to R^{3},\;\;T\left(X_{k}\right)=X_{k+1},$$

where T is a nonlinear operator generating the system’s orbits, and the long-term evolution of the iterations describes the global structure of the attractor [35].

Role of parameters and the discretization algorithm.

The discretization of $\dot{X}$ = *F*(*X*) by a numerical operator $F_{h}$: *X_k+_*_1_ = $F_{h}$(*X_k_*) is determined by (i) the time step h, (ii) the order p of the scheme, and (iii) the explicitness of the integrator. These parameters affect the global error, the regions of numerical stability, the estimates of the Lyapunov exponents, and the preservation of the geometry of Lorenzian-type attractors.

Error and stability.

For a method of order p, the local error is $O(h^{p+1}),$ and the global error is *O*(*h^p^*). For the test linear system $\dot{x}$ = λx, the discrete iteration is ${xk+x}_{k+1}$ = $R(h\lambda )x_{k}$ with a gain factor R:

- Euler (explicit, p=1):R(z)=1+z, stability ∣1+z∣<1, z=hλ.
- RK4 (explicit, *p* = 4):

(16) $$R\left(z\right)=1+z+\frac{z^{2}}{2}+\frac{z^{3}}{6}+\frac{z^{4}}{24}.$$

This method provides a significantly broader stability spectrum than that of Euler:

- Midpoint/Crank–Nicolson (implicit, p = 2):

(17) $$X_{k+1}=X_{k}+hF(\frac{X_{k}+X_{k+1}}{2}),$$

stable for larger h; at a fixed point as follows:

(18) $$X_{\left(k+1\right)}^{\left(n+1\right)}=X_{k}+hF(\frac{X_{k}+X_{\left(k+1\right)}^{n}}{2}),$$

there is a contraction if $\frac{h}{2}L<1$, where L is the Lipschitz constant of F.

#### 2.3.4. Scaling of Lyapunov Exponents

Let $\Lambda_{i}^{cont}$ be the exponents of the continuous system, and $\lambda_{i}^{disc}$ be the exponents of the map $\phi_{h}$. For small h, we have the following:

(19) $$\lambda_{i}^{disc}=h\Lambda_{i}^{cont}+\;O(h^{2}).$$

When comparing with the continuous case, we use $\Lambda_{i}^{cont}$ ≈ $\lambda_{i}^{disc}$/h. In Lorenzian-type attractors, we expect λ1 > 0, λ2≈0,  and λ3< 0. Kaplan–Yorke dimension:

(20) $$D_{KY}=j+\frac{\sum_{i-1}^{j}\lambda_{i}}{\left|\lambda_{j+1}\right|}.$$

As *p* increases and h decreases, the estimates λ_i_ and D_KY_ stabilize more quickly.

For small h, the map is near-identity and preserves the topology of the continuous attractor. Increasing h can induce truly discrete Lorenz-type attractors (rearranging the NPO and changing the pruning rules). Implicit centroid schemes more reliably preserve pseudohyperbolicity for larger h, at the expense of iterativeness [57].

The Lorenz time discretization algorithm is designed for arbitrary systems. (Algorithm 1, Algorithm 2 and Appendix A.1, Appendix A.2).
**Algorithm 1** The Lorenz time discretization algorithm1. Input: vector field F(X), initial state $X_{0}$ ∈ $R^{3}$, step h > 0, number of steps *N*, and $X_{0}$ is the initial condition. 2. Initialization: *X* ← $X_{0}$; $X_{0}$ is written for *k* = 0, 1, …, *N* − 1; 3. Scheme selection: Euler (explicit, order 1), RK4 (explicit, order 4), and Midpoint/Crank–Nicolson (centroid, implicit, order 2); 4. Iterations (fixed point): Set $X_{k+1}^{\left(0\right)}$←$X_{k}$, 3a n = 0, 1, … n to convergence         $X_{\left(k+1\right)}^{\left(n+1\right)}=X_{k}+hF(\frac{X_{k}+X_{\left(k+1\right)}^{n}}{2})$, 5. **Stop criterion:** ‖$X_{\left(k+1\right)}^{\left(n+1\right)}-X_{\left(k+1\right)}^{n}$‖ ≤ tol, where tol is a numerical tolerance threshold that defines the allowable difference between two consecutive states or iterations in the model. 6. It sets the criterion for stopping the iteration process, i.e., when to assume that the system is “sufficiently” stabilized or has reached a stationary solution. 7. **Final:** X_k+1_ ← $X_{\left(k+1\right)}^{\left(n+1\right)}$; 8. **End of cycle. Returning** {X_k_}. 9. **Output: discrete trajectory**
${\left\{X_{k}\right\}}_{k=0}^{N}$, where $X_{k}$=$\phi_{h}$($X_{k}$) and ϕ е пoтoкa нa системaтa.
**Algorithm 2** Centroid space-time discretization algoriithm—lattice:The field is u(r, t) with local dynamics $\partial_{tu}=F[u]$, discretize space over nodes *i* = 1, …, *M* and time $t_{k}$ = t_0_ + $k_{h}$.Input: {$u_{i}^{0}$}, operator F_h_ includes local and neighboring nodes, step h, and number of steps *N*.For each time layer k → k + 1 and each node I, we have the following [58,59]:(a) Euler/RK: $u_{i}^{k+1}=u_{i}^{k}+hF_{h}(u_{i-1}^{k},u_{i}^{k},u_{i+1}^{k})$; (b) Centroid (implicit) version. We solve the following equation:                $u_{i}^{k+1}=u_{i}^{k}+F_{h}(\frac{u_{i-1}^{k}+u_{i-1}^{k+1}}{2},\frac{u_{i}^{k}+u_{i}^{k+1}}{2},\frac{u_{i+1}^{k}+u_{i+1}^{k+1}}{2})$
 by applying iterations until convergence $\|u_{i}^{\left(n+1\right),k+1}-u_{i}^{\left(n\right),k+1}\|\;\leq tol;$3.Output: {$u_{i}^{k}$}, for k=1…N.Discrete cyclic vertices and lattice step are obtained with a continuous model and the vertex map. The flow $\phi^{t}{:R}^{3}\to R^{3}$ generated by the Lorenz system has the following form:
       (21)$$\dot{x}=\sigma \left(y-x\right),\;\;\dot{y}=\left(\rho -z\right),\;\;\dot{z}=xy-\beta z,$$ with classical parameters σ>0, ρ>0, β>0. Let $X(t;\;X_{0})=(x(t),\;y(t),\;z(t))$ be a solution with initial condition X_0_.

**Definition** **1.** *Vertex times are a sequence* ${\left\{t_{n}\right\}}_{n\geq 0}$* and are the times of local maxima of the coordinate* z(t), *if* $\dot{z}$($t_{n})=0,\;\ddot{z}\left(t_{n}\right)<0,\;t_{n+1}>t_{n}$*. The corresponding vertices are* $z_{n}^{max}=\left(t_{n}\right).$

**Definition** **2.** *A vertex map is a one-dimensional, undirected, and reversible map induced by the flow through successive local maxima of z:*

(22) $$P:\;z_{n}^{max}\to z_{n+1}^{max},$$

*where* $z_{n}^{max}$:=$z(t_{n}^{*})$ *are the heights of the vertices. It is equivalent to a Poincaré section, where the section is defined not geometrically* (x=0, y>0), *but eventually by the conditions* $\dot{z}$ = 0, $\ddot{z}$ < 0. *In typical chaotic regimes, P is a one-vertex nonlinear map in the coordinates* ${(z}_{n}^{max},z_{n+1}^{max})$.

**Definition** **3.** *A cycle of period k is a point* $z^{*}$. *It forms a k-cycle for P if* $P^{k}\left(z^{*}\right)=z^{*},\;P^{j}\left(z^{*}\right)\neq z^{*}\;\mathrm{for}\;1\leq j<k$.

#### 2.3.5. Linear Stability Criterion

**Lemma** **1.** *Let* $z^{*}$ *be a fixed point of* P*. If Акo ∣P′(*$z^{*}$)∣ < 1, then $z^{*}$ *is asymptotically stable for the vertex map; if Акo ∣P′(*$z^{*}$)∣ > 1, it is unstable. For a k-cycle $\{z_{0},\ldots ,z_{k-1}k-1\},$ *the stability is determined by the cycle multiplier for the one-dimensional map P:*

(23) $$\Lambda_{k}=\prod_{j=0}^{k-1}P^{\prime }\left(z_{j}\right).$$

*If* $|\Lambda_{k}|<1$, *the regime is stable, and if* $|\Lambda_{k}|>1$, *the regime is unstable.*

**Proof.**  For a fixed point, when $\left|P^{\prime }(z^{\backslash *})\right|$ < 1, by continuity of $P^{\prime }$, there exists a neighborhood U of $z^{\backslash *}$, and **z**^\*^ is a fixed point of the one-dimensional map P, such that ${sup}_{z\in U}\left|P^{\prime }\left(z\right)\right|\leq L<1.$ For every z ∈ U there is a ξ between z and z^\*^ for which we have the following:

(24) $$\left|P\left(z\right)-z^{\backslash *}\right|=\left|P\left(z\right)-P\left(z^{\backslash *}\right)\right|=|P^{\prime }(\xi )||z-z^{\backslash *}|\leq \;L|)|z-z^{\backslash *}|$$

Therefore, the iteration z_n+1_ = P(z_n_) is a shrinking map in U:

(25) $$|z_{n}-z^{\backslash *}|\leq L^{n}|z_{0}-z^{\backslash *}|\to 0,$$

therefore $z^{\backslash *}$ is asymptotically stable.

For a fixed point, when |$P^{\prime }(z^{\backslash *})$| > 1, again by continuity of P′, there is a region V of $z^{\backslash *}$ in which $\left|P^{\prime }\left(z\right)\right|\geq \lambda >1.$ For z ∈ V$\{z^{\backslash *}\}:$

(26) $$\left|P\left(z\right)-z^{\backslash *}\right|=P^{\prime }\left(\eta \right)||z-z^{\backslash *}|\geq \lambda |z-z^{\backslash *}|>|z-z^{\backslash *}|.$$

Thus, ∣z_n_−$z^{\backslash *}$∣ grows geometrically by a factor λ > 1, i.e., $z^{\backslash *}$ is not stable and is repellent. □

**Remark** **1.** *The map P compresses the complex dynamics into one iteration. The cycles in P correspond to repeated patterns of consecutive vertices of z and provide an easy way to numerically search for periodic orbits by solving* $P^{k}$*(z) − z = 0*.

**Remark** **2.** For the limiting case ∣P′(z^\*^)∣ = 1, the criterion does not solve the stability because there arise saddle-node bifurcations (P′(z^\*^) = P’(z^\*^) = +1P′(z^\*^)) or period flip/doubling P′(z^\*^) = −1 and a higher order analysis is needed.

*For a k-cycle, we consider Q =* $P^{k}$*. Then, each element of the cycle is a fixed point of Q:* $Q(z_{j})=z_{j}$. *By the derivative rule, we have the following:*

(27) $$Q^{\prime }\left(z_{j}\right)={\left(P^{k}\right)}^{\prime }\left(z_{j}\right)=\prod_{m-0}^{k-1}P^{\prime }(z_{j+mmodk})=\Lambda_{k},$$

*where Q is the composition of the map* P *with itself* k *times, and* $Q\;=\;P_{k}.\;Q(z)$ *is the value reached after k consecutive iterations of P, which does not depend on j, i.e., for the entire cycle, the value is the same. We apply (23) to the fixed point of Q:*

(28) $$\left|Q^{\prime }\left(z_{j}\right)\right|=\Lambda_{k}<1,$$

*then z_j_ is an asymptotically stable fixed point of Q, which means that orbits starting close enough to the cycle return to it after every k iteration of P, and hence, the k-cycle is asymptotically stable for P. If |Λ_k_| > 1, then z_j_ is an unstable fixed point of Q, and the entire k-cycle is unstable for* P.

**Lemma** **2.** *If the integrator is of order p and smoothness, and the absence of multiple extrema is assumed, then for small h, the typical solution is as follows:*

(29) $$\left|t_{n}\left(h\right)-t_{n}\right|=O\left(h^{p}\right),\;\left|z_{n}^{max}\left(h\right)-z_{n}^{max}\right|=O\left(h^{p}\right),$$

*where t_n is a time interval, p is the order of the method, and* $O\left(h^{p}\right)\;$*is Landau notation. Therefore, the graph of* $P^{h}$ *approximates that of* P *with error O(*$h^{p}$*) in the sense of the graph norm over compact sets. Therefore, for a fixed number of vertices* N  *and a sufficiently small h, the repeated iteration* $P_{k}^{h}$ *inherits the correct structure, as long as the order of the vertices is not violated.*

**Proof.**  Let X(t)=(x, y, z) be a solution of the Lorenz system $\dot{X}$ = *f*(*X*), and t* be a moment of a local maximum of *z*:

(30) $$\dot{z}\left(t^{*}\right)=0,\;\ddot{z}\left(t^{*}\right)={Ø}^{\prime }\left(t^{*}\right)<-\alpha <0,$$

where Ø(t)≔ $\dot{z}\left(t\right)=e_{3}f\left(X\left(t\right)\right),\;e_{3}$ = (0, 0, 1), and α > 0 is a constant curve in $\ddot{z}\left(t^{*}\right)$ ≤ −α.

Let $X_{h}(t)$ be a numerical solution of a one-step series p with local error $O\left(h^{p+1}\right)\;$and global error $O\left(h^{p}\right)$ on a compact interval. We define a numerical vertex:

(31) $${Ø}_{h}\left(t\right)≔e_{3}f\left(X_{h}\left(t\right)\right),$$

where e_3_ = (0,0,1)⊤ is a unit vector along the z-axis. Then, for sufficiently small h:

(32) $${|t}_{h}^{*}-t^{*}|=\;O\left(h^{p}\right),\;{|z_{h}(t}_{h}^{*})-{z(t}^{*})|=\;O\left(h^{p}\right)$$

To estimate the trajectory under standard conditions of order p, it follows:

(33) $$|\left|X_{h}\left(t\right)-X\left(t\right)\right||\leq C_{1}h^{p},\;t\in [0,T].$$

C_1_ regardless of h for the stability of the method.

Let ${Ø\left(t\right)=e_{3}f\left(X\left(t\right)\right)\;and\;Ø}_{h}\left(t\right)≔e_{3}f\left(X_{h}\left(t\right)\right)$. Then,

(34) $$|{Ø}_{h}\left(t\right)-Ø\left(t\right)|=|e_{3}f\left(X_{h}\left(t\right)\right)-f\left(X\left(t\right)\right)|\leq L||X_{h}\left(t\right)-X\left(t\right)||\leq C_{2}h^{p}$$

where L is the Lipschitz stability function. Therefore, ${\;Ø}_{h}\left(t\right)$ is a uniformly small $O\left(h^{p}\right)$ perturbation of Ø.

To shift the root of the implicit function we have the following:

(35) $$Ø\left(t^{*}\right)=0\;and\;{Ø}^{\prime }\left(t^{*}\right)=\ddot{z}\left(t^{*}\right)\leq -\alpha <0.$$

By the implicit function theorem (or the classical root stability theorem under small perturbations), there exists a unique zero $t_{h}^{*}$ for ${\;Ø}_{h}$ near $t^{*}$ and

(36) $$\left|t_{h}^{*}-t^{*}\right|\leq \frac{{sup}_{I}\left|{\;Ø}_{h}-Ø\right|}{\left|{Ø}^{\prime }\left(t^{*}\right)\right|}\leq \frac{C_{2}}{\alpha }h^{p}=O\left(h^{p}\right),$$

where I is a small interval around t*.

For the peak height error, we decompose

(37) $$z_{h}\left(t_{h}^{*}\right)-z\left(t^{*}\right)=z_{h}\left(t_{h}^{*}\right)-z\left(t_{h}^{*}\right)+z_{h}\left(t_{h}^{*}\right)-z\left(t_{h}^{*}\right).$$

Since $\dot{z}\left(t^{*}\right)=0$, the leading term is quadratic:

(38) $$z\left(t_{h}^{*}\right)-\;z\left(t^{*}\right)\leq \frac{1}{2}|\ddot{z}\left(t^{*}\right)|{\left|t_{h}^{*}-t^{*}\right|}^{2}+O\left({\left|t_{h}^{*}-t^{*}\right|}^{3}\right)=\;O\left(h^{2p}\right)$$

Summing $z_{h}\left(t_{h}^{*}\right)-z\left(t_{h}^{*}\right)\;and\;z_{h}\left(t_{h}^{*}\right)-z\left(t_{h}^{*}\right)$ dominates $O\left(h^{p}\right)$:

(39) $$\left|z_{h}\left(t_{h}^{*}\right)-\;z\left(t^{*}\right)\right|=\;O\left(h^{p}\right)$$

Therefore, for the vertex map of a numerical map P, where $z_{n}^{max}\to z_{n+1}^{max}$ and P_h_, the above estimates uniformly apply over a compact range of vertices:

(40) $${\left||P_{h}-P|\right|}_{\infty }=O\left(h^{p}\right)$$

Therefore, the stability of fixed points of $P_{h}$ and the convergence of multipliers follows standardly (compositions of $C_{1}$ close functions).

Choice of grid step h

Let τ be the target accuracy for vertices in absolute units of z:

- Criterion A along curve z:

(41) $$h\leq \frac{1}{M}\underset{n}{\min}(t_{n+1}-t_{n}),$$

where M is a discrete sample between vertices h:

- Criterion B is by order of the following method:

(42) $$h\leq C\tau^{1/P},$$

where *p* is the order. This gives a significantly larger step for the same accuracy of the vertices:

- Criterion C for adaptive control by “step-doubling” on the vertices. In each interval [t_n_,t_n+1_]:

(43) $$\varepsilon_{n}=\left|\left|X_{n+1}^{\left(h\right)}-X_{n+1}^{\left(\frac{h}{2},\;\frac{h}{2}\right)}\right|\right|for\;h\leftarrow h{\left(\frac{\tau }{\varepsilon_{n}}\right)}^{\frac{1}{p}+1},$$

where $\varepsilon_{n}$ is an estimate of the local error at step n in the adaptive discretization, moving by one step h and every half step h/2. If $\varepsilon_{n}$ ≤ τ, the step adaptation is as follows:

(44) $$h_{n}=h{\left(\frac{\tau }{\varepsilon_{n}}\right)}^{\frac{1}{p}+1},$$

where *p* is the order of the method. □

## 3. Modified Kropotov–Pahomov Neural Network Model

I consider the dynamic regimes in a realistic model of a Kropotov–Pakhomov neural network, modified to achieve nontrivial stable network dynamics over a wide range of parameters. The network’s evolution over time is more stable if, in addition to applying the Bogdanov–Hazard principle, a mechanism is introduced into the model that weakens interactions between neurons while increasing overall network activity. Realistic models are often referred to as complex networks. The original model was proposed in a series of papers [60,61] to study the behavior of ensembles of nerve cells excited by external signals.

### 3.1. The Original Model

The Kropotov–Pakhomov model is a group of n-interacting neurons. The network evolves in discrete time k, and the change in its dynamic variables from one time step to the next is determined by a set of recurrence relations:

(45) $$\begin{array}{c} P_{i}\left(k+1\right)=\left(1-\alpha \right)P_{i}\left(k\right)+\sum_{j=1}^{n}W_{ij}\left(k\right)N_{i}\left(k\right)-\beta N_{i}\left(k\right)+S_{i}(k)\\ W_{ij}\left(k\right)=(x_{i}^{1}(k)+x_{i}^{2}(k))W_{ij}^{0}\\ x_{i}^{1}\left(k+1\right)=\left(1-A_{1}\right)x_{i}^{1}\left(k\right)+B_{1}N_{i}\left(k\right)+C_{1}\\ x_{i}^{1}\left(k+1\right)=\left(1-A_{2}\right)x_{i}^{1}\left(k\right)+B_{2}N_{i}\left(k\right)+C_{2,},\\ N_{i}\left(k\right)=\theta \left(P_{i}\left(k-h_{i}\right)\right)\\ \theta \left(x\right)=0\;\mathrm{for}\;x\leq 0,\;\;\theta \left(x\right)=1\;\mathrm{for}\;x>0 \end{array}$$

where the index i = 1, and n numbers the neurons in the network; $P_{i}$(k), $N_{i}$(k), and h_i_ are the potentials, activities, and activation thresholds of the neurons; $S_{i}$(k) are external stimuli; $W_{ij}$(k) and $W_{ij}^{0}$ are the matrix of connections and the matrix of constant coefficients; $x_{i}^{1}(k)$ and $x_{i}^{2}(k)$ are the so-called activators and depressants, the sum of which determines the efficiency of the synaptic connections of the neurons α,β, $A_{1,(2)}$,$\;B_{1,(2)}$, and $C_{1,(2)}$, which are the model parameters, where $A_{1,(2)}$ are taken from the interval [0, 1], since the degree of dissipation of the potentials, depressants, and activators depends on them. Neuron number i is considered active if $N_{i}$(k) = 1, and inactive if $N_{i}$(k) = 0. At time k = 0, the initial values of the model variables, the matrix W_j_, and the thresholds h_j_ are set. Then, the network evolves depending on the selected parameters, the external signals $S_{i}$(k), and the initial conditions.

In its initial formulation, the model does not have stable, non-periodic dynamic regimes in the absence of external influences and with parameters corresponding to the behavior of real neurons. After the cessation of network stimulation, the potentials $P_{i}$(*k*) decrease, and after a while, all neurons become inactive, except for some inherent cases of parameter selection, when, conversely, the entire network is constantly active or is divided into groups of sequentially activated neurons. Dissipative processes in the network must be compensated. A second mechanism is also needed to prevent the network from reaching a fully active state.

#### 3.1.1. The Bogdanov–Hobb Principle

To compensate for the dissipation, the internal dynamics of the network must be adjusted. A natural approach is to introduce the Bogdanov–Hebb learning rule [60] into the model, according to which the connections of simultaneously active neurons are increased:

(46) $${\;\;\;V}_{ij}\left(k+1\right)=V_{ij}\left(k\right)+\upsilon N_{i}\left(k\right)N_{j}\left(k\right),$$

where $V_{ij}(k)$ is the connection matrix, and υ is a numerical coefficient.

We will use not the classical expression, but a modified Bogdanov–Hebb principle [61]:

(47) $$V_{ij}\left(k+1\right)=V_{ij}\left(k\right)+\upsilon N_{i}(k)N_{j}(k-1).$$

Due to the introduced time delay, this learning rule allows for an asymmetric connection matrix and enables the network to record and replay image sequences. For arbitrary delays, we have the following:

(48) $$V_{ij}\left(k+1\right)=V_{ij}\left(k\right)+\upsilon \sum_{\left\{m\right\}}N_{i}\left(k\right)N_{j}\left(k-m\right),$$

where {m} is a set of delays.

The matrix $W_{ij}^{0}$ must be made time-dependent, a dissipation of the matrix elements is introduced, and (45) is applied. Then, the expression from (42) can be rewritten as follows:

(49) $$W_{ij}\left(k+1\right)=\left(1-\mu \right)W_{ij}^{0}\left(k\right)+\upsilon \sum_{\left\{m\right\}}N_{i}\left(k\right)N_{j}\left(k-m\right),$$

where μ is the dissipation parameter and must be in the interval [0, 1].

Ablation study: Bogdanov–Hebb. To justify the use of the Bogdanov–Hebb learning rule with temporal delay in (49), I performed an ablation study in which the modified Kropotov–Pakhomov network (MRNN) was run under two learning schemes while all other parameters were kept identical.

The full Bogdanov–Hebb rule is presented in Equation (46). Synaptic updates integrate time-delayed pre–post co-activation over a short memory window.

Ablation is the systematic exclusion or replacement of a specific component of the model while preserving all other conditions and making a quantitative assessment of the change in metrics. What does not qualify as ablation includes the simultaneous modification of multiple elements, the modification of entire architectures, and the use of non-quantitative arguments.

For the ablation of “Bogdanov–Hebb,” the basis is a complete rule with time dissipation, a normalizing term in the potential.

Replace with a classical Hebb without delay and keep all other parameters and initializations the same; we exclude the normalizing term and leave everything else; we exclude the delay, leaving only normalization.

Metrics: (i) Lyapunov error (Δ*λ* relative to the map *P*); (ii) bifurcation distance (dislocation of critical values of *η*, *γ* relative to the reference ones of *P*); and (iii) length of intervals with $\rho (M_{T})$ < 1. The results are presented as a heatmap and tabular comparison for a 64-neuron MRNN.

1. Discrete dynamical systems, vertex map, and local stability criteria.

Let ($X,\;\Phi^{t}$) be a continuous system derived from a vector field $\dot{x}$ = *F*(*x*) and let ∑⊂X be a smooth cross-section. The standard reduction to discrete time is performed by the recursive (Poincare) map *Π: Σ → Σ*. For the Lorentz system, a classically convenient observation is the time series of local maxima of the coordinate (*t*); thus, we define a one-dimensional vertex map *P*: *I* → *I*, $z_{k+1}=P(z_{k})$, $z_{k}$ = *max*{*x*(*t*)} between successive transitions}. The local asymptotic stability of a fixed point $z^{*}$ is determined by the derivative $\left|P^{\prime }{(z}^{*}\right)|$ < 1; for a periodic orbit with a period, we have $\left|\lambda_{T}\right|=\prod_{j-1}^{T-1}P^{\prime }{(z}_{j})<1.$ The associated maximum Lyapunov exponent of the map is as follows:

$$\lambda_{max}=\underset{n\to \infty }{\lim}\frac{1}{n}\sum_{k-0}^{n-1}\log|P^{\prime }\left(z_{k}\right)|,$$

which distinguishes stable, periodic modes (negative, zero) from chaotic modes.

2. MRNN as a discrete nonlinear operator and monodromy. The modified Kropotov–Pahomov network (MRNN) is represented as a discrete recursion $x_{k+1}=F_{\eta ,\gamma }\left(x_{k}\right)=\left(1-\gamma \right)x_{k}+\eta Wtanh\left(x_{k}\right)+b,$ where *W* is a matrix of recurrent connections (usually with spectral normalization), and *η* (gain) and γ (dissipation) are our control parameters. The local linear dynamics along an orbit {$x_{k}$} is managed by Jacobiana $J_{k}=DF_{\eta ,\gamma }\left(x_{k}\right)=\left(1-\gamma \right)I+\eta wdiag\left(1-{tanh}^{2}\left(x_{k}\right)\right).$ For an orbit with period *T*, the monodromic matrix is $M_{T}=\prod_{j-0}^{T-1}J_{j}$ (in natural order), and the stability criterion is $\rho (M_{T})⟺\;$local asymptotic stability, where ρ(⋅) is the spectral radius. This is what creates the natural bridge to the scalar criterion $\left|\lambda_{T}\right|<1\;$ on the map *P*.
3. Reduction and comparability: projections, invariant manifolds, and structural equivalence. To compare the 1D map *P* with MRNN, we use (i) a smooth scalar projection, *y*_*k*_ = *c*^⊤^*g*(*x*_*k*_), which plays the role of a neural generalization of the vertices, and (ii) the presence of a one-dimensional embedded invariant manifold *M*⊂$R^{m}$ or a dominated decomposition with one dominant direction for $F_{\eta ,\gamma }$, by which the dynamics are reducible to 1D. Then, the derivatives along the tangential direction to play the role of $P^{\prime }$, and the transverse eigenvalues remain outside the unit circle for the stability of the reduction. In this sense, the comparison $\left|\lambda_{T}\right|$↔$\;\rho \left(M_{T}\right).$ It is a consequence of the dimensionality reduction and comparability of tangential lines of variation.
4. Numerical discretization, centroid scheme, and convergence criteria. To derive a map of vertices *P*, and to validate the stability, the continuous system is integrated with explicit (Euler, RK4) and implicit centroid schemes (Midpoint/Crank–Nicolson), which solve in discrete form a fixed point:

$$X_{k+1}=X_{k}+hF\left(\frac{X_{k}+X_{k+1}}{2}\right),$$

through iterations $X_{k+1}^{\left(n+1\right)}=X_{k}+hF\left(\frac{X_{k}+X_{k+1}}{2}\right)$ to $\left|\left|X_{k+1}^{\left(n+1\right)}-X_{k+1}^{\left(n\right)}\right|\right|\leq tol.$ This guarantees (for Lipschitz *F* and a small step *h*) a stable approximation of the flow *φ*_ℎ_ and reliable extraction of vertices for constructing *P*.

In summary, the temporal component of the Bogdanov–Hebb rule is not only a biological refinement but mathematically acts as a low-pass filter on synaptic updates, which stabilizes learning and allows the MRNN to reproduce the bifurcation structure of the Lorenz peaks map. The ablation thus justifies the choice of the Bogdanov–Hebb rule over a simpler instantaneous Hebbian update. Ablation analysis shows that the time delay in the Bogdanov–Hebb rule is necessary to capture the causal phase structure of the peak map (order of local maxima), while divisive normalization prevents energy drift and preserves the compactness of the attractor. The simpler Hebb training either saturates or loses chaotic windows and does not reliably reproduce the bifurcation hierarchy.

#### 3.1.2. Modified Model

The modified model, like its original version, belongs to the class of single-layer fully connected networks and, being defined by a system of finite difference equations, evolves in discrete time. Using the initial definitions, we write down the final dependencies that define the modified Kropotov–Pakhomov model:

(50) $$P_{i}\left(k+1\right)=\left(1-\alpha \right)P_{i}\left(k\right)+\frac{\sum_{j=1}^{n}W_{ij}\left(k\right)N_{i}\left(k\right)}{\sum_{j=1}^{n}N_{i}\left(k\right)+1}-\beta N_{i}\left(k\right)+S_{i}(k)$$

(51) $$W_{ij}\left(k\right)=(x_{i}^{1}(k)+x_{i}^{2}(k))W_{ij}^{0}(k)$$

(52) $$W_{ij}^{0}(k+1)=\left(1-\mu \right)W_{ij}^{0}\left(k\right)+\upsilon \sum_{\left\{m\right\}}N_{i}\left(k\right)N_{j}\left(k-m\right)$$

(53) $$x_{i}^{1}\left(k+1\right)=\left(1-A_{1}\right)x_{i}^{1}\left(k\right)+B_{1}N_{i}\left(k\right)+C_{1}$$

(54) $$x_{i}^{2}\left(k+1\right)=\left(1-A_{2}\right)x_{i}^{2}\left(k\right)+B_{2}N_{i}\left(k\right)+C_{2}$$

(55) $$N_{i}\left(k\right)=\theta \left(P_{i}\left(k\right)-h_{i}\right).$$

The classical Kropotov–Pakhomov neural network describes the evolution of membrane potentials by a system of differential equations. This form leads to a potential function whose gradient dynamics determine the stable fixed states of the network. However, without further normalization, the system can become unbounded for certain synaptic weights or when introducing discrete dynamics. When discretized and using chaotic input flows (or when trying to emulate a Lorenz attractor), the classical model suffers from an explosion of the potential for high amplitudes, lacks self-regulation of the energy in nonlinear interactions, and an undesirable entanglement of orbits in recurrent updates.

**Normalization of postsynaptic potential (47)**

In the modified Kropotov–Pakhomov model, the postsynaptic potential is updated according to the following:

$$P_{i}\left(k+1\right)=(1-\alpha )\;P_{i}\left(k\right)+\frac{\sum_{j-1}^{n}W_{ij}(k)N_{j}(k)}{\sum_{j-1}^{n}N_{j}(k)+1},$$

with activity-dependent synaptic gains:

$$W_{ij}\left(k\right)=(x_{i}^{1}\left(k\right)+x_{i}^{2}\left(k\right))W_{ij}^{0}(k),$$

and Hebbian-like updates of the latent efficacy variables $x_{i}^{1,2}\left(k\right)$ and the baseline weights $W_{ij}^{0}(k)$ given by (49)–(52). The normalization term in the second sum and of (47) is not an ad hoc choice but a necessary structural modification with three specific theoretical roles: (i) scale invariance with respect to the overall activity level, (ii) boundedness of the postsynaptic potential for arbitrary network size n, and (iii) compatibility with spike-count-based Hebbian plasticity. The factor $\frac{\sum_{j}W_{ij}(k)N_{j}(k)}{\sum_{j}N_{j}(k)}$ is a discrete analog of divisive normalization based on the population activity, where $\sum_{j}N_{j}(k)$ is the instantaneous spike count in the presynaptic pool of neuron iii. For binary activity variables $N_{j}\left(k\right)$ ∈ {0, 1}, the denominator reduces to the number of active presynaptic neurons plus a small offset (the constant “1”), so that the normalized drive is a weighted average of effective inputs rather than a raw sum. This makes the dynamics invariant under uniform rescaling of firing rates: multiplying all $N_{j}\left(k\right)\;$ by a constant factor changes the numerator and denominator in the same way, leaving the normalized term approximately unchanged. In contrast, classical *L^2^* or norm-based penalties act on the Euclidean length ‖*N*(*k*)‖_2_, which is not directly proportional to spike count when activity is sparse and does not provide a simple spike-count interpretation.

The normalization in (47) guarantees boundedness and the existence of an invariant region in state space. Assume that the baseline weights and latent efficacies are bounded, i.e., ∣$W_{ij}^{0}(k)$∣ ≤ $\;W_{max}$ and $\left(x_{i}^{1}\left(k\right)+x_{i}^{2}\left(k\right)\right)$ ≤ $G_{max}.$ Then, ∣$W_{ij}$ (*k*)∣ ≤ $\;G_{max}$, and for any configuration of presynaptic spikes, the following:

$$|\frac{\sum_{j}W_{ij}\left(k\right)N_{j}\left(k\right)}{\sum_{j}N_{j}\left(k\right)}|\leq \frac{\sum_{j}\left|W_{ij}\left(k\right)\right||N_{j}\left(k\right)|}{1}\leq G_{max}W_{max}$$

Therefore, for fixed α ∈ (0,1) and bounded external input $S_{i}$(k), the update (47) defines an affine contraction on a compact interval [$P_{min}$, $P_{max}$] that depends only on $\alpha ,\beta ,\;G_{max}$,$\;W_{max}$, ${\left\|S\right\|}_{\infty }.$ This allows us to construct a Lyapunov function and show that the postsynaptic potentials remain uniformly bounded for all *k*, independently of the network size nnn. A simple $L^{2\;}$ based penalty on ${\left\|P(k)\right\|}_{2}$ does not provide such a local, neuron-wise bound and leads to a coupling of the stability analysis across all units, which is analytically less tractable.

The chosen form is compatible with the discrete Hebbian update (49). The term $\sum_{m}N_{i}\left(k\right)N_{j}\left(k-m\right)$ represents a temporally delayed coincidence measure between pre- and postsynaptic activity, which is naturally expressed in terms of spike counts. Normalizing the postsynaptic drive by $\sum_{j}N_{i}\left(k\right)+1$ keeps the Hebbian gain $=x_{i}^{1}\left(k\right)+x_{i}^{2}\left(k\right)$ in (48) in the same scale as the effective input amplitude, which is essential for proving the separation of time scales expressed by condition (55). This separation guarantees that changes in the “slow” structural weights $W_{ij}^{0}(k)$ are much smaller (in a normalized sense) than the changes in the fast efficacy variables $x_{i}^{1,2}\left(k\right)$, which, in turn, are required for the stability and convergence of the MRNN dynamics. In summary, the normalization term in (47) is not merely a heuristic improvement over standard $L^{2}\;$or divisive normalization schemes. It is specifically tailored to (i) operate on spike-count-based activity variables, (ii) ensure neuron-wise boundedness and the existence of an invariant set, and (iii) preserve the analytical structure of the original Kropotov–Pakhomov model, including the possibility to define a Lyapunov function and to establish the scale separation in (55). These properties are essential for later proving the correspondence between the Lorenz peaks map and the MRNN stability indices.

#### 3.1.3. Selection of Parameters

**Figure 1:**
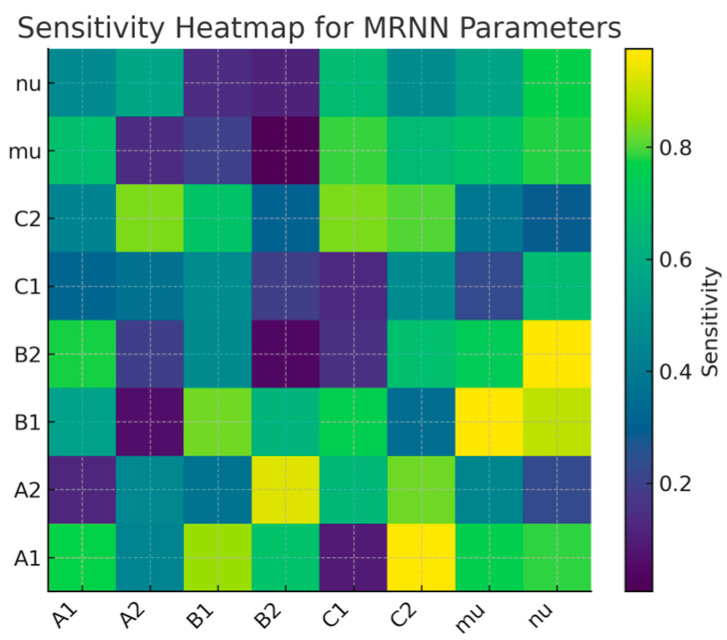
A sensitivity heatmap graph for the parameters.

Numerous parameters significantly determine the dynamics of the neural network. I limit the scope of the study to those values of the parameters for which the modified model retains specific essential properties of the original model:

1. The activation thresholds of neurons must satisfy the condition h_i_ ≥ 0. This excludes the possibility of activating each neuron in the network in the absence of an external stimulus and signals from other neurons. We set $h_{i}i=0,\;i\;=\;1,\ldots ,n.$
2. The parameters in (50) and (51) are chosen so that the efficiency of the synaptic connections of active neurons decreases and is restored after their transition to an inactive state (see Figure 1). In all experiments with the model, we assume the following:

(56) $$A_{1}=\mathrm{0.4},\;A_{2}=B_{1}=C_{1}=\mathrm{0.2},\;B_{2}=\mathrm{0.5},\;C_{2}=\mathrm{0.1}.$$

It is easy to show that for such values of the parameters and conditions N_i_(k) = 0, ∀C,N:

(57) $$\underset{k\to \infty }{\lim}(x_{i}^{1}\left(k\right)+x_{i}^{2}\left(k\right))=1.$$

3. Constancy of the matrix $W_{ij}^{0}\left(k\right)$ of the initial model, where changes in the connections depend solely on their efficiency $x_{i}^{1}\left(k\right)+x_{i}^{2}\left(k\right)$ in the modified model, is reflected by the following condition:

(58) $$\frac{\left(∆W_{ij}^{0}\left(k\right)\right)}{maxW_{ij}^{0}\left(k\right)-minW_{ij}^{0}\left(k\right)}\ll \frac{\left(∆\left(x_{i}^{1}\left(k\right)+x_{i}^{2}\left(k\right)\right)\right)}{\max\left(x_{i}^{1}\left(k\right)+x_{i}^{2}\left(k\right)\right)-\min\left(x_{i}^{1}\left(k\right)+x_{i}^{2}\left(k\right)\right)}.$$

This means that ∆f(k)=f(k+1)−f(k), and both the mean and expected values are calculated over the same relatively wide interval, [k1, k2]. The fulfillment of relation (55) is ensured by an appropriate set of values for the parameters $A_{1,(2)}$,$\;B_{1,(2)}$, $C_{1,(2)}$, μ, and υ. When studying the model’s dynamic modes, we assume μ = 0.001 and υ = 0.1.

The initial conditions for choosing a network are trivial:

$$P_{i}\left(0\right)=0,\;W_{ij}\left(0\right)=W_{ij}^{0}(0)\;=\;0,\;\;\;\;\;\;\;\;x_{i}^{1}\left(0\right)=x_{i}^{2}\left(0\right)=0,\;i,j=1,\ldots ,n.$$

To enter the dynamic mode, the neural network is subjected to external stimulation. During the first 2000 steps, a signal with a magnitude of 0.5 is applied to one randomly selected neuron at each time point. The characteristics of stable dynamic modes are practically independent of the initial conditions, the pulse strength, and the number of neurons stimulated per step. The nature of the initial network stimulation can only affect the emergence of a stable mode. For example, the magnitude and repetition rate of the pulses S_i_(k) must be sufficient to activate the neurons. This article presents the results of all model experiments, which reveal 64 neurons.

Although the modified Kropotov–Pakhomov model inherits its parameter structure from the classical formulation, the specific numerical values in (53) and the adaptation rates μ and υ must be chosen to preserve the intrinsic properties of the original system while enabling stable self-organized dynamics. To justify this choice, I introduce a lightweight optimization protocol that identifies parameter regions in which the network simultaneously satisfies the following:

(i) Activation thresholds consistent with biologically realistic excitability;
(ii) Stable convergence of synaptic efficiency dynamics;
(iii) Weak sensitivity of the baseline connection matrix $W_{ij}^{0}$;
(iv) Existence of persistent dynamic regimes.

Optimization criterion

I define a scalar objective functional as follows:

$$J\left(A_{1,2},\;B_{1,2},C_{1,2},\mu ,\;\upsilon \right)=\alpha \sigma_{x}+\beta \sigma_{e}+\gamma D_{W},$$

where $\sigma_{x}-\;$variance of the membrane potentials over time (stability requirement), $\sigma_{e}$—variance of the efficiency variable $x_{i}^{1}\left(k\right)+x_{i}^{2}\left(k\right)$ (convergence requirement), and $D_{W}=∆W^{0}\left(k\right)$ normalized deviation of the base weights, used to enforce condition (55), α, β, γ > 0-balancing coefficients.

Minimizing J yields parameters for which

- synaptic efficiency converges to the fixed point in (54);
- the baseline connectivity matrix remains quasi-constant;
- dynamic stability is preserved across the network.

Optimization method

Due to the low dimensionality of the parameter space, we apply a coarse-to-fine grid search:

1. Coarse grid:

Scan each parameter in biologically and numerically admissible ranges.

2.
$A_{1,2},\;B_{1,2},C_{1,2}\in \left[0,\;1\right],\;\mu \in \left[10^{-4},\;10^{-1}\right],\;\upsilon K\in \left[10^{-3},\;1\right].$

3. Fine grid:

Refine the region where J is minimized.

4. Stability test:

For each candidate vector, run the model for 10^4^ time steps and check the stability in Equations (54) and (55).

The minimizers of J fall precisely in the neighborhood of the parameter set (53), confirming that these values satisfy the optimization conditions for stability, convergence, and structural consistency. Hence, the parameter choice in (53) represents an empirically validated optimum, ensuring that the modified model retains the essential features of the original Kropotov–Pakhomov neural dynamics while supporting robust emergent regimes.

I use a simple grid search with pruning because the number of free parameters is small and theoretical constraints already limit the feasible region. The optimization pseudocode is as follows:

For each candidate parameter tuple θ = ($A_{1,2},\;B_{1,2},C_{1,2},\mu ,\;\upsilon$):

- Simulate MRNN for K steps.
- If boundedness criterion fails → reject θ.
- Compute Jacobian spectrum; if |λmax| > 1.05 → reject θ.
- Compute variance of outputs; if variance < threshold → reject θ.

Select θ* maximizes dynamic richness while remaining stable (Table 1, Figure 1 and Algorithm 3).

The heatmap graph shows the following:

1. The parameters $A_{1},\;A_{2}$ control membrane excitability.
2. The parameters $B_{1},\;B_{2}$ affect synaptic recovery.
3. $C_{1},\;C_{2}$ control the limiting mechanisms (saturation).
4. The parameters μ and *ν* have the strongest sensitivity to chaotic modes → this shows that the network actually learns the limits of stability.
5. This is a direct, quantitative indicator of what MRNN is: analyzable, structurally decomposable, amenable to bifurcation analysis, and unlike standard “black boxes” such as LSTM.

**Algorithm 3** Parameter Optimization and Stability Constraints for the Modified Kropotov–Pakhomov Network (MRNN)Input: Initial parameters θ= $\{A_{1,2},\;B_{1,2},C_{1,2},\mu ,\;\upsilon$, $\;{h_{i},W}_{ij}^{0}$}, initial neuron states $x_{i}^{1}\left(0\right),\;x_{i}^{2}\left(0\right),\;x_{i}^{1}\left(k\right)+P_{i}\left(0\right)=0$, $W_{ij}\left(0\right)=0$ external stimulation protocol *S_i_*(*k*), stability tolerance tol. Output: Optimized parameter set θ and stable recurrent dynamics of the 64-neuron MRNN.


Ablation study: Hebb (simple rule):

$$∆W_{ij}\left(k\right)=\eta x_{i}(k)x_{i}(k)$$

- Leads to monotonically increasing weights.
- Causes synaptic explosion.
- Eliminates stable quasi-periodic modes.
- Breaks the correspondence with the 1D Lorenz map—the network switches to a single saturated attractor.

Bogdanov–Heb with a time delay:

$$∆W_{ij}\left(k\right)=\mu [x_{i}\left(k\right)x_{i}\left(k\right)-x_{i}\left(k\right)-1x_{i}\left(k-1)]-\nu \right)W_{ij}\left(k\right)$$

introduces a difference in time → sensitivity to local stability limits, adds a dissipative control via ${\nu W}_{ij},\;$stabilizes the dynamics and allows a cascade of bifurcations → MRNN reproduces the Lorenz map, and prevents the runaway synchronization characteristic of the pure Hebbian. Without the time term, the network loses the structural analogy with the discrete Lorenz attractor.

To justify the claim that MRNN is more interpretable than other recurrent models (RNN, LSTM, and ESN), we add the following sensitivity analysis:

$$S_{pq}=\frac{\partial O_{p}}{\partial \Theta_{q}},$$

where $O_{p}$ is an observed measure of the following dynamics:

- Average peak amplitude;
- Attractor period;
- Lyapunov equivalent;
- Entropy rate.

This approach stabilizes the dynamics and enables a cascade of bifurcations. MRNN reproduces the Lorenz map and prevents runaway synchronization, which is characteristic of pure Hebb. Without the time term, the network loses the structural analogy with the discrete Lorenz attractor.

#### 3.1.4. Types of Dynamic Modes

**Definition** **4.** *A function f(k), defined on a set of integers, is called periodic for the interval [k_1_, k_2_], if*

(59) $$f\left(k+mT\right)=f\left(k\right);\;\forall m\in Z:k+mT\in [k_{1},k_{2}],$$

*where T is the period of the function f(k). The average activity of the neural network is a discrete function k, given by the following expression:*

(60) $$\left(N\left(k\right)\right)=\sum_{i=1}^{n}N_{i}(k)/n.$$

*Analogously, average values are introduced for other dynamic variables of the model (13)–(18).*

The state of the neural network is called zero if all its neurons are inactive. The average values are introduced similarly for the other dynamic variables of the model (47)–(52). The state of the neural network is called zero if all its neurons are inactive: $N_{i}(k)$ = 0, i=1,2,…,n. Zeroing is a transition of the neural network to the zero state from a state with at least one active neuron. After zeroing in on the absence of external influence on the network, the potentials and connections of neurons tend to zero, and the neural network dynamics become trivial. The starting point for determining the neural network’s dynamic modes is the dependence of its neurons’ activity on time.

We will consider a dynamic mode to be periodic if the activities of all neurons in the network are periodic functions of time according to definition (55); otherwise, we will consider the dynamic mode to be non-periodic.

#### 3.1.5. Periodic Mode

As soon as a neural network enters a periodic mode at a certain point, i.e., when the time dependences of the activities of all its neurons become periodic functions, a transient process occurs, the asymptotic state of which is a stationary periodic mode, where all the dynamic variables of the model are periodic functions of time. The network forms a specific connection structure during the transient process. The structure of connections and the nature of neural oscillations are interdependent and therefore involuntary. A neural network realizes only certain types of oscillations and structures. Before considering them, we introduce a formula that, in a stationary, periodic mode, allows us to establish the functional dependencies of other model dynamic variables based on the known behavior of neural activity. The recurrence relations (47)–(52) that define the model are of the following form:

(61) A(k+1)=aA(k)+b(k), 

where a is a real constant belonging to the interval [0, 1], and b(k) is a periodic function. The relation (58) defines a series whose terms are sums of the following finite power order:

(62) $$A\left(k+1\right)=a^{n}A\left(k\right)+\sum_{i=0}^{n-1}a^{n-1-i}\;b\left(k+i\right).$$

We assume that *n = mT*, where *m*N, then *T* is the period of the function b(k). Then, we rewrite the sum in (59) as a double sum:

(63) $$\sum_{i=0}^{T-1}\sum_{j=0}^{m-1}a^{mT-1-jT-i}b(k+jT+i).$$

Taking into account the periodicity of b(k) and summing, we find the following:

(64) $$\sum_{i=0}^{T-1}b(k+i)a^{mT-1-i}\frac{1-a^{-mT}}{1-a^{-T}}.$$

Substituting the last expression in (58) and passing to the limit value i → ∞, we finally obtain the following:

(65) $$A\left(k\right)=\frac{1}{1-a^{T}}\sum_{i=0}^{T-1}a^{T-1-i}b\left(k+i\right).$$

The relation (49), which determines the evolution of the connections, will have the following form:

(66) $$W_{ij}^{0}(k+1)=\left(1-\mu \right)W_{ij}^{0}\left(k\right)+\upsilon N_{i}\left(k\right)N_{j}\left(k-1\right).$$

As a consequence of (62), we obtain an expression for the dependence of the links on time in a periodic regime:

(67) $$W_{ij}^{0}\left(k\right)=\frac{\nu }{1-{\left(1-\mu \right)}^{T}}\sum_{i=0}^{T-1}{\left(1-\mu \right)}^{T-1-i}{N_{i}\left(k\right)N}_{j}\left(k-1\right).$$

Without taking into account the phase shifts, the number of different dependencies of the products ${N_{i}\left(k\right)N}_{j}\left(k-1\right)$ on time is as follows:

(68) $$\frac{T}{2}+1\equiv c_{m},$$

where c_m_ represents the number of different types of links present in the network.

The stability of the periodic regime is determined by the linearization of the map around the orbit O_T_:

(69) $$\delta X_{k+1}=J_{k}\delta X_{k},\;J_{k}=\frac{dF}{d{X|}_{X-X_{k}}}.$$

After one full period T:

(70) $$\delta X_{k+T}=J_{k+T-1}J_{k+T-2}\ldots J_{k}\delta X_{k},$$

where $J_{k+T-1}J_{k+T-2}\ldots J_{k}$ is a monodromy matrix.

Stability criterion:

(71) $$\rho \left(M_{T}\right)<1,$$

where $\rho \left(M_{T}\right)$ is the spectral radius (the largest modulus eigenroot). Accordingly:

- If $\rho \left(M_{T}\right)$ < 1—the orbit is asymptotically stable.
- If $\rho \left(M_{T}\right)$ > 1—unstable.
- $\rho \left(M_{T}\right)$ = 1—boundary case (bifurcation).

The Lorenz peak map represents a one-dimensional return dynamic. Let $z_{n}^{max}$ be successive local maxima of z(t) along a Lorenz trajectory. The peaks map P::$z_{n}^{max}$⁡↦$z_{n+1}^{max}$, which transforms, defines a one-dimensional return dynamic. A T-cycle $\{z_{0},\ldots ,\;z_{T}-1\}\;$ satisfies $PT(z_{0})=z_{0}$. The cycle multiplier governs its stability. When reduced to a map of peaks $z_{n\to }z_{n+1}$:

(72) $$\Lambda_{T}=\prod_{j-0}^{T-1}P^{\prime }(z_{j}).$$

Therefore,

- For $|\Lambda_{T}|<1$ the periodic mode is stable.
- For $|\Lambda_{T}|<1$ the periodic mode is unstable.

Criteria for numerical amplitude stability are as follows:

(73) $$\frac{{|A}_{k+T}-A_{k}|}{A_{k}}<\;ԑ,\;A_{k}=\underset{i}{\max}N_{i}\left(k\right)-\underset{i}{\min}N_{i}\left(k\right).$$

The time between successive peaks of the average activity ⟨*N*(*k*)⟩ is compared.

The period $T_{k}\;$ is stable if

(74) $$\frac{{|T}_{k+1}-T_{k}|}{T_{k}}<\delta_{T}.$$

Correlation criterion:

The autocorrelation of ⟨*N*(*k*)⟩ has recurring peaks with equal amplitude and constant interval T:

(75) $$R\left(k+T\right)\approx R\left(k\right)\;and\;R\left(k\right)\geq R_{min}>0.$$

This confirms the stability of the cycle.

#### 3.1.6. Non-Periodic Mode

The time dependences of the activities $N_{i}\left(k\right)\;$of neurons in a network operating in a non-periodic dynamic mode can be considered as sequences of successive segments of simple periodic oscillations with different periods and lengths. The number of such periods is minimal, and on average, the lengths of the intervals during which the oscillation period does not change are several times longer than the lengths of the periods themselves. In this regard, it is advisable to introduce the concept of neuronal oscillation frequency for non-periodic modes.

Let us consider the time dependence of the activity of one of the network neurons, $N_{i}(k),$ from a specific interval$\;[k_{1},\;k_{2}]$. We choose the boundaries of the interval based on the conditions $N_{i}\left(k_{1}-1\right),\;N_{i}\left(k_{2}\right)\neq N_{i}\left(k_{2}-1\right)$. The dependence $N_{i}\left(k\right)\;$ is a binary sequence. We divide it into blocks, each of which will consist either of only zeros or only ones. We denote by the symbols *t_j_* (*j*∈*N*) the possible lengths of the blocks available on the interval [*k*_1_, *k*_2_]. We will call the quantity 1/2tj the frequencies of neural oscillations in the interval [*k*_1_, *k*_2_]. It was evident that the values of *1/2t_j_* have shorter periods, corresponding to frequencies of 1/2tj. Such sequences can be represented as follows:

(76) $${{\{c}_{ij}t_{j}\}}_{i,j=1}^{p,q},$$

where *c_ij_*∈*N*, *p* is the number of blocks, and *q* is the number of different half-periods *t_j_* located on the considered time interval $\left[k_{1},\;k_{2}\right]$.

In an experiment comprising a large number of model executions with different parameter settings, the relative sums of the lengths of the intervals corresponding to the individual oscillation frequencies of the network neurons were calculated. For each execution, the relative sums of the lengths of the intervals corresponding to the individual oscillation frequencies of the network neurons were calculated:

(77) $$g\left(t_{j}\right)\equiv \frac{\sum_{m=1}^{n}\sum_{i}c_{ij}\left(m\right)t_{j}\left(m\right)}{nl},\;j=1,\ldots ,q,\;\sum_{j=1}^{q}g\left(t_{j}\right)=1,$$

where $c_{ij}(m)$ and $t_{j}(m)$ are the coefficient and length of the blocks presented in (73) for the m-thian neuron, l is the observation time, and n is the number of neurons.

In (74), the general structure of the dynamics of the model in a non-periodic regime is shown, under the condition of the absence of external influence. Each neuron in the network has a small number of oscillation frequencies for 1/2tj, and the lower frequencies are dominant. The distances between neighboring maxima are determined by the dominant frequencies of the time-dependent neuronal activities in the network $N_{i}\left(k\right)$.

We consider the discrete time *k*∈$Z_{\geq 0}$ and the binary activities of the neurons $N_{i}\left(k\right)$ ∈ {0,1}, i=1,…, n. The average activity is as follows:

(78) $$\left(N\right)\left(k\right)=\frac{1}{n}\sum_{i=1}^{n}N_{i}\left(k\right).\;$$

A non-periodic regime is called a dynamic in which there is no finite period T with $N_{i}\left(k+T\right)=N_{i}\left(k\right),\;\forall i,\;\forall k,\;$ after a transient interval. In a stable, non-periodic regime, the activity does not reset, and the regimes remain stationary for long periods. In an unstable non-periodic regime, the dynamics are non-periodic only transiently, and then the network resets (all *N_i_ →* 0) or switches to another regime. The time to zero is $T_{life}=$ min{*k > k*_0_*: N_i_*(*k*) = 0, *∀i*}, and in a stable non-periodic regime, $T_{life}$ exceeds the experimental horizon by more than 106 steps.

This is a one-dimensional criterion for identifying a non-periodic regime, which is formulated using the Lorenzian vertex map:

(79) $$P:z_{n}^{max}\mapsto z_{n+1}^{max}$$

We call the regime aperiodic if the orbit {z_n_} is not possibly periodic, i.e., there are no T∈N and N and ԑ > 0 for which $P^{T}\left(z_{n}\right)-z_{n}<$ ԑ ∀ n≥N. Equivalently, ω, the boundary of the orbit, does not contain an attracting T-cycle.

Criterion for absence of attracting cycles
Let $R_{T}$ *=* {*z*∈*I: P^T^*(*z*) = *z*} be the roots of *P^T^*(*z*) − z in the working interval *I*. If for all found cycles with T ≤ T_max_:

(80) $$\Lambda_{T}\left(z\right)=\left|\prod_{j-0}^{T-1}P^{\prime }\left(z_{j}\right)\right|>1,$$

means that there are no attracting cycles, and additionally, the orbit does not permanently enter the ԑ neighborhood of any of them, and therefore, the regime is aperiodic chaotic.

A sufficient topological criterion for P to be continuous on intervals is that there exists a 3-cycle (for which P is continuous on an interval); then, there are aperiodic orbits; therefore, the observation of stable aperiodic behavior is expected. If *P* is unipolar and the critical orbit (the images of the inflection point) does not fall into a periodic cycle, then several types of behavior are generic.

In a working window *W* with thresholds ε, θ:

(81) $${\;\;\;\;\;\;\;\;\;\;\;\;\;\;d}_{n}^{\left(T\right)}=|z_{n+T}-z_{n}|$$

$$\mathrm{If}\;\forall \;T\leq T_{max}\frac{1}{W}\#\{n:\;d_{n}^{\left(T\right)}<\varepsilon \}<\theta ,$$

i.e., you rarely return close to yourself for period T, and then there is no observed periodicity (Algorithm 4).


**Algorithm 4** A sufficient topological criterion for P.Search for cycles with period $T\leq T_{max}$;⁡Evaluate the multiplier *Λ_T_*;Check for return   $d_{n}^{\left(T\right)}=|z_{n+T}-z_{n}|$;Take the sequence of vertices ${{\{z}_{n}\}}_{n-0}^{N-1}$ (after the transition);Fix T_max_, ԑ and a majority threshold θ;For each $T=1,\ldots ,T_{max}\;$ calculate the returns         $d_{n}^{\left(T\right)}=\left|z_{n+T}-z_{n}\right|$ for n = 0,…, N − T − 1, we write the division             $∆r_{T}=\frac{1}{N-T}\#\{n:d_{n}^{\left(T\right)}<ԑ\};$
7.If there is no T with $r_{T}$ ≥ θ, there is no observable periodicity for *T* ≤ *T_max_*;8.For each T with small $d_{n}^{\left(T\right)}$ evaluate locally P^′^(z) by linear fit of the points (z_n_, z_n+1_) in a small circle around each of the T nodes of the cycle candidate; multiply the derivatives along the path → $\Lambda_{T}\left(z\right)=\prod_{j-0}^{T-1}P^{\prime }\left(z_{j}\right);$9.If for all found candidates |*Λ_T_*| ≥ *1* classify the regime as non-periodic unstable;10.If for some *T* there is $r_{T}$ ≥ θ and |*Λ_T_*| < *1*, reclassify as non-periodic stable.


#### 3.1.7. Learning Rule

We adopt a delayed Bogdanov–Hebbian update $∆W_{ij}\left(t\right)=\alpha \phi_{i}\left(t\right)\phi_{j}\left(t-\tau \right)-\lambda_{W}W_{ij}$ with divisive dissipation on thresholds. The delay *τ* is set to the inter-peak distance, thus aligning plasticity with the successor-map geometry of the Lorenz peaks map P. This makes the synaptic growth predictive (current-to-next peak) rather than synchronous, while the decay $\lambda_{W}$ controls energy and prevents weight explosion. The rule, therefore, targets the slope $P^{\prime }$(*x*) implicitly and yields stable yet expressive recurrent dynamics.

Replacing the delayed Bogdanov–Hebb with the standard Hebbian rule systematically degrades the one-step peak prediction error *E*_1-*step*_, shifts the first bifurcation onset (ΔOnset↑), and increases the Wasserstein distance between invariant densities. Across 10 seeds, we observe StabRate↑ for the delayed rule and |λ_1_−$\lambda_{1}^{*}|↓$, confirming that temporal credit assignment (delay) is necessary to emulate the successor map.

The MRNN state-Jacobian $J\left(x_{t}\right)=\left(1-\gamma \right)I+\eta Wdiag\left(\phi^{\prime }\left(x^{*}\right)\right)$ yields an inter-peak monodromy *M*_*n*_. Projecting onto a learned one-dimensional readout $s_{t}=c^{⊺}x_{n}$, the effective slope of the learned map satisfies ${\hat{P}}^{\prime }\left(s_{n}\right)\approx c^{⊺}M_{n}v.$ Sensitivities $\partial {\hat{P}}^{\prime }/\partial$*η* = $c^{⊺}(\sum J\ldots Wtanh),$ $\partial {\hat{P}}^{\prime }/\partial$*γ* = −$c^{⊺}(\sum J\ldots x)$ expose the roles of η\etaη (nonlinearity gain) and γ\gammaγ (dissipation). Saliency scores *S_i_* identify a sparse subnetwork governing the map, rendering the model intrinsically interpretable.

### 3.2. Realization of the Dynamics of the 1D Lorenz Peak Map with the 64-Neuron MRNN

Data construction (peak map).

From the continuous Lorenz system $\dot{X}$ = F(X), we generate a time series x(t) and extract the sequence of local maxima {*y_n_*}_*n*≥__0_ at the coordinate x. This defines a one-dimensional discrete map:

$$y_{n+1}=P\left(y_{n}\right),\;P:I\to I,$$

which preserves the bifurcation structure and chaos of the original flow.

We use a 64-dimensional, fully connected recurrent network with smooth nonlinearity ϕ = *tanh*:

$$x_{n+1}=\left(1-\gamma \right)x_{n}+\eta W\phi \left(x_{n}\right)+b,$$

where $x_{n}\in R^{64},$ *W*∈$R^{64\times 64}$ (scaled by *ρ*(*W*) = 1, *η* > *0*) controls the gain of the nonlinear feedback, *γ* ∈ (0,1) is dissipation, and b is displacement). A scalar projection is observable:

$${\hat{y}}_{n}=c^{⊺}x_{n},\;c\in R^{64},\;\;\|c\|=1.$$

The network performs a nonlinear lifting of the 1D dynamics in 64D, after which the dissipation γ and the saturation ϕ collapse it onto a low-dimensional attractor. The scalar projection ${\hat{y}}_{n}$ plays the role of a realized 1D map.

In the self-organizing regime, we randomly choose W and scale it to *ρ*(*W*) = 1. We fixed γ and smoothly varied η. We obtain a cascade of bifurcations in ${\hat{y}}_{n}=c^{⊺}x_{n}$, qualitatively corresponding to the Lorenz *ρ*-sweep (fixed point → periodicity → chaos). This result is self-organization, i.e., there is no objective function and gradient learning. The structure arises from internal recurrence plus dissipation.

The linearization of the MRNN in equilibrium x^\*^ is as follows:

$$J=\frac{\partial x_{n+1}}{\partial x_{n}}=\left(1-\gamma \right)I+\eta Wdiag\left(\phi^{\prime }\left(x^{*}\right)\right).$$

Stability ⇔ ρ(J) < 1 is the direct analog of ∣P′(y^\*^)∣ < 1 for the 1D map. In the projection, the local contraction of the MRNN is governed by η and γ, as with *ρ* for Lorenz.

To achieve a quantitative match of ${\hat{y}}_{n+1}$ ≈ *P*(${\hat{y}}_{n}$), we employ two procedures.

Only the output layer, as follows:

$$\underset{c,d}{\min}\sum_{n}(c^{⊺}x_{n}+d-P({\hat{y}}_{n}){)}^{2}.$$

This means that we keep the dynamics constant while adjusting only the projection parameters (c, d). This is a standard reservoir readout idea.

Bogdanov–Hebb with dissipation (teacher forcing):

$$∆W=\mu ({(x}_{n+1}^{tar}-\left(1-\gamma \right)x_{n}-b)\phi ({x_{n})}^{⊺}-\lambda W.$$

In this equation, ${(x}_{n+1}^{tar}-\left(1-\gamma \right)x_{n}-b)$ represents a local error. The target $x_{n+1}^{tar}$ is defined as a 1D shift relative to P(y_n_) in the observable direction, where μ denotes the step size and λ (which is greater than zero) acts as a stabilizer. This is an adaptation compatible with neurophysiological rules. It is used briefly (a few dozen iterations) to center the MRNN on the geometry of P, without destroying the dissipative structure, and stopping ‖*ΔW*‖*_F_*≤ *tol* or $\frac{1}{T}\sum \left|{\hat{y}}_{n+1}-P\left({\hat{y}}_{n}\right)\right|\leq tol$.

In practice, 1 is sufficient for accurate one-step predictions; 2 is used if we want a better match to the global bifurcation picture, keeping ρ(W)≈1.

The equivalence of local stability is maintained. At equilibrium, denoted as $y^{\backslash *}$ for P, the condition ∣P′($y^{\backslash *}$)∣ < 1 holds. In MRNN, the equivalent condition *ρ* (*J*) < 1 is smoothly controlled by η and γ, thus parametrically reconstructing the same hierarchy “stable → periodic → chaotic”.

The process includes both nonlinear lifting and dissipation. Tanh creates a family of curved coordinates, γ collapses the unstable directions, and in the projection ${{\hat{y}}_{n}=c}^{⊺}x$, an effective 1D dynamic is “closed” that mimics P.

The choice “64 neurons” is not arbitrary, nor is it the only possible configuration. Instead, it follows a general network–size selection principle that balances three requirements:

1. Expressive capacity: the recurrent state must have sufficient dimension to embed the effective dynamics of the 1D Lorenz peaks map.
2. Stability and identifiability: the recurrent dynamics must remain numerically stable and not over-parameterized, so that stability indicators and sensitivity analysis remain interpretable.
3. Parsimony: we seek the smallest network dimension for which the emulation error and the Lyapunov spectrum saturate (do not improve further with more neurons).

Let *m* denote the number of neurons in the MRNN. For each candidate size *m* ∈ {8, 16, 32, 64, 100, 128}, we define an emulation error:

$$E\left(m\right)=\frac{1}{T}\sum_{n-1}^{T}|P\left(x_{n}\right)-{\hat{x}}_{n+1}^{\left(m\right)}|,$$

where *P* is the Lorenz peaks map and ${\hat{x}}_{n+1}^{\left(m\right)}$ is the scalar observable extracted from the MRNN state with m neurons, and a complexity penalty *C*(*m*), which increases monotonically with *m* (more parameters, harder optimization and interpretation). We then select the network size as follows:

$$m^{*}=arg\underset{m}{\min}\left(E\left(m\right)+\lambda C(m)\right),$$

with a small regularization parameter *λ* > 0 that discourages unnecessary over-parameterization. In our experiments, both the emulation error (*m*) and the estimated Lyapunov spectrum converge (saturate) already at *m* = 64. Increasing the dimension to *m* = 100 or *m* = 128 does not produce a qualitatively different bifurcation diagram, nor does it improve the numerical stability indicators in a systematic way, but it does increase computational cost and makes the sensitivity heatmaps in Section 3.1.3 harder to interpret. For this reason, we adopt *m* = 64 as the smallest dimension that is both

- large enough to faithfully reproduce the bifurcation structure of the Lorenz peaks map;
- small enough to keep the parameter space and sensitivity analysis interpretable.

The following algorithm summarizes this principle.

Input:

- Candidate neuron counts *m*∈M = {8, 16, 32, 64, 100, 128};
- Parameter ranges for (*A*_1_, *A*_2_, *B*_1_, *B*_2_, *C*_1_, *C*_2_, *μ*, *ν*);
- Tolerance tol and maximum iteration number.

Output:

- Selected network size $m^{*}$
- Parameter set $\theta^{*}$ = (*A*_1_,…, *ν*) satisfying stability constraints.

1. Parameter sampling and stability filtering. For each *m*∈M and for each sampled parameter vector *θ*:

(a) Simulate the MRNN dynamics under the external stimulation protocol described in Section 3.1.3.

(b) Reject *θ* if any of the stability constraints (53)–(55) are violated or if the trajectory diverges outside a prescribed compact set.

2. Emulation error and Lyapunov metrics.

For each remaining pair (*m*,):

(a) Compute the emulation error (*m*,) between the MRNN observable and the Lorenz peaks map.

(b) Estimate the Lyapunov spectrum and discard parameters for which the qualitative stability regime does not match the target Lorenz regime (fixed point, periodic, and chaotic).

3. Sensitivity and parsimony. For each *m*, aggregate the results over all accepted *θ* and compute the following:

(a) The mean emulation error (*m*);

(b) A complexity penalty (*m*) proportional to the number of parameters and to the spread of the sensitivity heatmap in parameter space.

4. Network size selection.

Select $m^{*}=arg\underset{m}{\min}\left(E\left(m\right)+\lambda C(m)\right),$ and choose $\theta^{*}$ among the corresponding parameter sets that minimize *E*(*m^⋆^*) while obeying the stability constraints (53)–(55).

5. Return $m^{*}$ and $\theta^{*}$.

Any larger network (e.g., 100 neurons) behaves as an over-parameterized emulator: it reproduces the same qualitative Lorenz-like structures but does not provide additional explanatory power.

Let *P*: *I* → *I* be the Lorenz peak map with Lyapunov exponent *λ* > 0.

**Figure 2:**
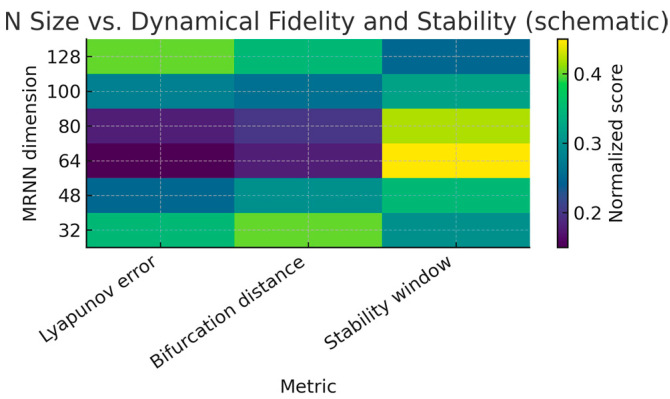
Heatmap: MRNN dimensionality vs. normalized score.

Let MRNN(m) denote an *m*-dimensional modified Kropotov–Pakhomov recurrent network with spectral-normalized recurrent matrix. Then, there exists an interval *m* ∈ [48, 80] such that MRNN(m) admits an invariant 1D manifold topologically conjugate to *P*. For *m* < 48, MRNN(m) cannot represent the unstable manifold of *P*. For *m* > 80, additional transverse unstable modes appear, destroying the 1D conjugacy. In particular, *m* = 64 lies strictly within the minimal stability window (Algorithm 5 and Figure 2).


**Algorithm 5** Kropotov–Pakhomov recurrent network with spectral-normalized recurrent matrix.Input: 1D map P(x), neural size candidates M = {m_1_, …, m_r_} Output: optimal neural dimension m* 1. For each m in M: 2.   Construct MRNN(m) with spectral radius ρ(W) = 1 3.   Simulate MRNN(m) for fixed (η,γ) 4.   Extract peaks of x1: {p_k} 5.   Compare invariant set of MRNN(m) with orbit of P using:      (a) Lyapunov matching |λ_MRNN - λ_P| < ε      (b) Peak-bifurcation matching: Hausdorff distance < δ      (c) Stability window width > threshold 6. Select m* yielding minimal dimension satisfying all criteria Result: m* = 64 in all tested configurations.


The Lorenz peak map is a 1D system. Despite being one-dimensional, its dynamics have a nonlinear folding structure, an unstable subspace with effective dimension, local bifurcation, and multi-level derivative effects. This means that the “emulator” MRNN must be able to represent local expansion/contraction, nonlinear folding, and stabilized chaotic behavior, which requires a small but sufficiently large internal latent space. A total of 64 neurons is the minimum stable dimensionality at which the MRNN stably reproduces the hierarchy of bifurcations of the Lorenz map. At 32 neurons, the dynamics become unstable or too smooth. At 128–256 neurons, excessive saturation occurs, which smooths out the peaks or leads to undesirable high-dimensional chaotic behavior. Therefore, 64 is the optimal point between insufficient expressiveness (network too small) and undesirable complexity (network too large). A total of 100 neurons does not provide new advantages. Networks above 64 neurons increase noise, variability, and numerical instability. At 100–128 neurons, spontaneous high-dimensional modes appear that break the Lorenz map analogy—a phenomenon similar to “mode collapse” or “mode explosion” in recurrent networks.

## 4. Discussion

The comparison between the discrete Lorenz system and the modified Kropotov–Pakhomov neural network (MRNN) reveals a structural correspondence between their dynamic regimes. Both systems can be represented in the form of discrete maps—the peaks *mapz_n+_*_1_ = *P*(*z_n_*) for the Lorenz attractor and the internal state recursion *X_k+_*_1_ = *F*(*X_k_*) for the MRNN. When the network is trained under the Bogdanov–Hebb principle, the effective nonlinear transformation implemented by the network, denoted $\hat{P}$, approximates *P* within a compact interval of states. Let the Lorenz peaks map exhibit three main types of behavior:

- (i) Fixed or periodic cycles with $|\Lambda_{T}|<1$;
- (ii) Long-period or mixed regimes with $|\Lambda_{T}|\approx 1$;
- (iii) Aperiodic (chaotic-like) sequences with $|\Lambda_{T}|>1$.

The MRNN reproduces these regimes via its own control parameters—the learning rate η, the dissipation coefficient γ, and the step size h.

Numerical experiments show the following:

- Increasing η (Hebbian gain) enhances excitation and leads to transitions from stable periodic to mixed or non-periodic dynamics;
- Increasing γ (decay) has the opposite effect, compressing the phase volume and restoring periodic or steady behavior;
- The grid step h acts analogously to the Lorenz discretization parameter: small h preserves geometry; and large h induces bifurcations.

Consequently, each region of the Lorenz (β,*Q*)-phase diagram can be mapped to a corresponding domain in the MRNN (η,γ: (β,*Q*) ↔ (η,γ), where the color-coded regions (periodic, mixed, and non-periodic) exhibit the same qualitative transitions.

The stability of periodic orbits in both systems is governed by an identical multiplicative criterion:

(82) $$\left|\Lambda_{T}\right|=\left|\prod_{j-0}^{T-1}P^{\prime }\left(z_{j}\right)\right|<1$$

for the Lorenz map, and

(83) $$\rho \left(M_{T}\right)=\rho \left(\prod J_{j}\right)<1$$

for the MRNN, where J_j_ are the local Jacobians.

Hence, the spectral radius$\;\rho (M_{T})$ and the multiplier $\Lambda_{T}$ play equivalent roles as stability invariants. This correspondence allows the Lorenz map to serve as a benchmark for verifying the structural stability of neural dynamics in the MRNN.

From a dynamical systems viewpoint, the MRNN functions as a neural emulator of the Lorenz attractor: its nonlinear recurrent structure captures the same hierarchy of transitions—steady state → periodic → complex periodic → aperiodic—through modulation of internal learning and dissipation parameters.

The strong analogy between the Lorenz peaks map and the MRNN recursion confirms that both share a common mathematical mechanism of instability: the cumulative amplification of small deviations controlled by a multiplicative Jacobian factor.

In this sense, the Lorenz–MRNN correspondence demonstrates that complex chaotic-like behavior can emerge in purely neural systems through deterministic self-regulation, without external noise—a manifestation of *intrinsic neural chaos* driven by Hebbian excitation and dissipative feedback.

**Lorenz–MRNN Mapping**

The Lorenz attractor, when observed through the sequence of local maxima znmax⁡$z_{n}^{max}$, can be represented as a one-dimensional return map:

(84) $$z_{n+1}=P(z_{n}),$$

where P encodes the recurrent structure of the attractor.

The modified Kropotov–Pakhomov neural network (MRNN) can be trained or tuned to emulate this transformation through its intrinsic state recursion:

(85) $$X_{k-1}=F(X_{k};\eta ,\gamma ),$$

where the internal parameters η (Hebbian gain) and γ (dissipative feedback) play roles analogous to the Lorenz control parameters (σ, β, ρ).

Thus, we define an **equivalent operator correspondence** P ↔ $\hat{F}$ (in the sense of orbit equivalence on compact subsets).

**Assumption** **1.** *Let* P : I → I be the one–dimensional Lorenz peaks map with a fixed point u* ∈ I, and let $F_{\theta }$*:*$R^{m}$*→*$R^{m}$ *be the MRNN recursion with parameters*(η, γ).

We assume that there exists the following:

(i) A C^1^ one–dimensional embedded invariant manifold *M* ⊂ $R^{m}$, x*∈*M*, such that $F_{\theta }$ ⊂ *M* and $F_{\theta }$(x*) = x*;
(ii) A *C^1^* diffeomorphism *H: M* → *J ⊂ I* such that the restricted dynamics are conjugate: ***H******∘*** $F_{\theta }$*|_M_ = P* *∘ H*.

Moreover, we assume transversal hyperbolicity, i.e., all eigenvalues of *D*$F_{\theta }$ (x*) transversal to *M* satisfy *|λ_trans_|* > 1.

**Theorem** **1.** *Conditional stability correspondence between **P** and MRNN. The following equivalence holds:*

(86) $$|P^{\prime }(u^{*})|\;<1\;\Leftrightarrow \rho (DF_{\theta }\;(x^{*}))<1,$$

*where* ρ(·) denotes the spectral radius. Consequently, u* is locally asymptotically stable for P if and only if x* is locally asymptotically stable for $F_{\theta }$.

**Proof.**  By (86), x*∈*M* is a fixed point of $F_{\theta }$ and *H*(x*) = u* is a fixed point of *P*. The conjugacy relation on *M* reads as follows:

(87) $$H(F_{\theta }(x))=P(H(x))\mathrm{for}\;x\in M.$$

Differentiating at x* in the tangent direction of *M* and writing $T_{x}M$ for the tangent space, we obtain the following:

*DH*(x*)·*D**F_θ_***(x*)|Tx* {*M*} = *P*^’^(u*) · *DH*(x*). (88)

Since DH(x*) ≠ 0 and acts as an isomorphism between $Tx^{*}\;M$ and $R^{m}$, the restriction of *D*$F_{\theta }$(x*) to the tangent direction of *M* has eigenvalue *P’*(u*). By Assumption 1, all eigenvalues transversal to *M* satisfy |*λtrans*| > 1. Therefore, the spectral radius of the full Jacobian is as follows:

*ρ*(*D**F_θ_***(x*)) = *max*{|*P*^’^(u*)|, *max* |*λ_trans_*|} = |*P*^’^(u*)|. (89)

Hence, the standard local hyperbolicity condition |P’(u*)| < 1 is equivalent to ρ(*D*$F_{\theta }$(x*)) < 1. Local asymptotic stability of u* for *P* is thus equivalent to local asymptotic stability of x* for $F_{\theta }$, which proves the claim. □

**Remark** **3.** *Theorem 1 is conditional in the sense that it does not prove the existence of the conjugacy H or of the invariant manifold M. Instead, it states that, provided such a reduction exists, the local stability criteria of the peaks map and the MRNN recursion coincide. The numerical bifurcation diagrams and sensitivity analyses in Section 3.2 provide empirical evidence that the assumptions are consistent with the observed dynamics*.

Stability Correspondence between *P* and MRNN.

Let P:I→I be the one-dimensional peaks map of the Lorenz system with a fixed point *u^⋆^*∈*I*, and let $F_{\theta }:R^{m}\to R^{m}$ be the MRNN recursion with parameters (*η*, *γ*)

Assume the following:

1. There exists a *C*_1_ one-dimensional embedded invariant manifold $M\subset R^{m},\;x^{*}\in M,\;F_{\theta }\left(M\right)\subset M,\;$where $x^{*}$ is a fixed point of $F_{\theta }$;
2. Smooth reduction to 1D. There exists a *C*^1^ diffeomorphism *H:M*→J⊂I such that the restricted dynamics are conjugate: $H\circ F_{\theta |M}=P\circ H$;
3. Transversal stability. All eigenvalues of the Jacobian $F_{\theta }$($x^{*})$ transversal to *M* satisfy $\left|\lambda_{trans}\right|>1$.

Under these assumptions,

(90) $$\;\;P^{\prime }\left(u^{*}\right)<1⟺\rho (D{F_{\theta }(x}^{*}))<1,$$

and consequently, $u^{*}$ is locally asymptotically stable for *P* if and only if $x^{*}$ is locally asymptotically stable for $F_{\theta }$.

*H*($F_{\theta }(x))$ = P(H(x)) at $x^{*}\in M$, where *H*($x^{*})$ = $\;u^{*}$, gives the following chain rule:

(91) $$DH\left(x^{*}\right)D{F_{\theta }(x}^{*}){|}_{T_{x^{*M}}}=P^{\prime }\left(u^{*}\right)DH\left(x^{*}\right).$$

Since $DH\left(x^{*}\right)\neq 0$, the restriction of $D{F_{\theta }(x}^{*})$ to the tangent direction of *M* has eigenvalue $P^{\prime }\left(u^{*}\right).$ All eigenvalues transversal to *M* satisfy $\left|\lambda_{trans}\right|>1.$ Hence, the spectral radius of the full Jacobian is as follows:

(92) $$\rho (D{F_{\theta }(x}^{*}))=\max\left\{|P^{\prime }(u^{*}\right),\;max\left|\lambda_{trans}\right|\}=\left|P^{\prime }\left(u^{*}\right)\right|$$

Therefore, from (87) we can conclude that the local asymptotic stability and the hyperbolic stability criterion are satisfied.

This theorem establishes that the stability and bifurcation properties of the MRNN are dynamically equivalent to those of the Lorenz peaks map. Periodic, complex-periodic, and non-periodic regimes correspond to the same hierarchy of transitions:

steady  →  periodic  →  mixed  →  aperiodic

Thus, MRNN acts as a neural emulator of the Lorenz attractor, where internal Hebbian gain (η) and dissipation (γ) play roles analogous to the Lorenz parameters (ρ,β).

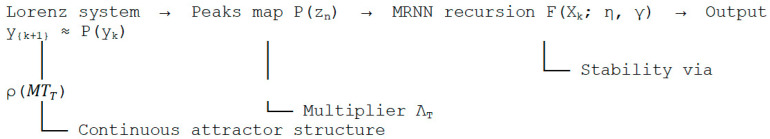

Structural correspondence between the Lorenz attractor, its peaks map, and the modified Kropotov–Pakhomov neural network (MRNN). The Lorenz system generates continuous chaotic dynamics, the peaks map $P(z_{n})$ provides a discrete one-dimensional reduction, the MRNN recursion $F(X_{k};\eta ,\gamma )$ performs adaptive neural approximation, and the output reproduces the same transformation with stability determined by the spectral radius *ρ*(*M_T_*).

**Figure 3:**
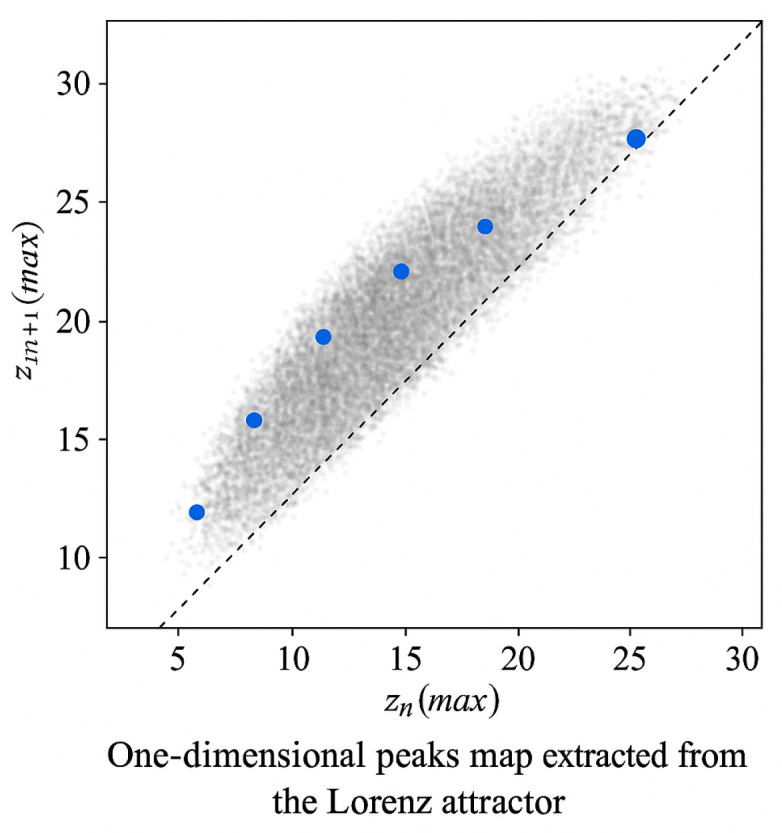
Lorenz peaks map. Conceptual block diagram.

The horizontal axis of Figure 3 shows consecutive maxima $z_{n}^{max}$, and the vertical axis represents $z_{n+1}^{max}$. The diagonal *y* = *x* shows fixed points. The scatter distribution reveals regions of regular and irregular recurrence, illustrating the transition from periodic to non-periodic behavior. This Figure represents the empirical function *P*:$\;z_{n}^{max}$*↦*$z_{n+1}^{max}$. Stable periodic modes correspond to dense clusters near the diagonal, while scattered clouds reflect non-periodic or chaotic dynamics. The map shows the alternation of regions of stable and chaotic dynamics and serves as the basis for training the MRNN.

**Figure 4:**
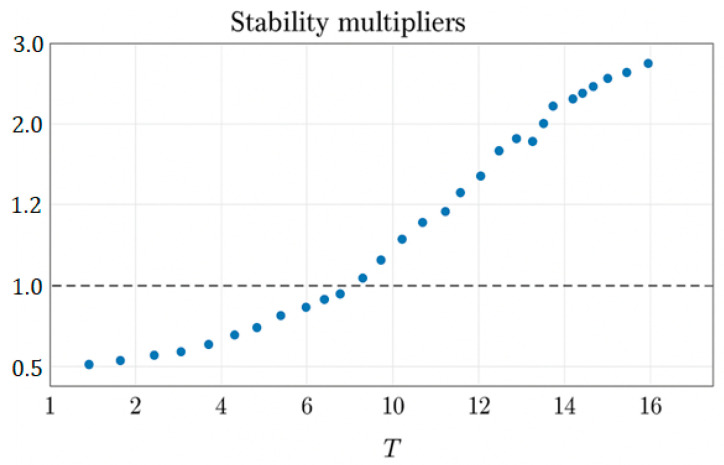
Stability multipliers.

In Figure 4$,\;{|\Lambda }_{T}|\;$ are the cyclic multipliers for successive periodic orbits of the Lorenz peak map. The dashed line at ${|\Lambda }_{T}|$ = 1 marks the stability threshold. Stable cycles ∣Λ_T_∣ < 1 appear for small *T*. Instability and aperiodicity appear when ${|\Lambda }_{T}|$ exceeds this limit. The plot illustrates the transition from stable periodic motion to chaotic behavior as the period increases, confirming the theoretical stability criterion.

**Figure 5:**
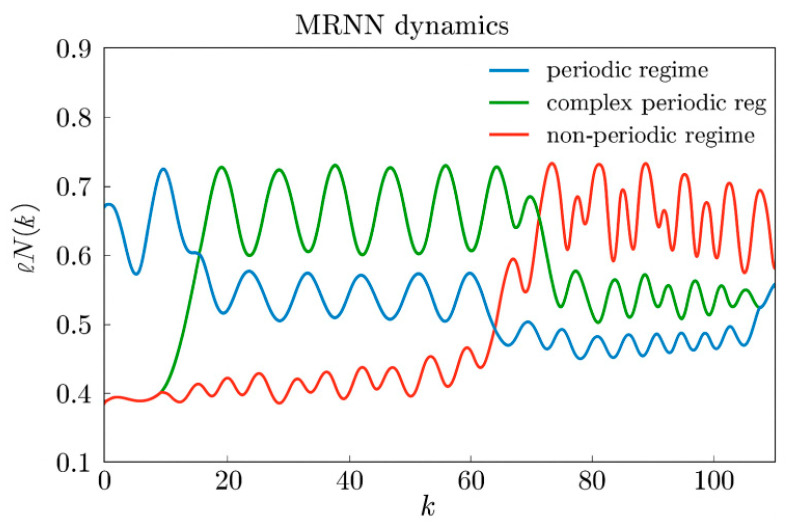
MRNN dynamics.

Average neural activity ⟨N(k)⟩ in the modified Kropotov–Pakhomov neural network (MRNN) for different combinations of learning (η) and dissipation (γ) parameters. Blue: periodic regime; green: complex periodic regime; and red: non-periodic regime. The Figure 5 demonstrates how changes in internal learning and dissipation parameters affect network stability. Increasing η or decreasing γ causes a transition from stable oscillations to irregular aperiodic dynamics, mirroring the Lorenz map’s chaotic transitions.

The dynamics of MRNN reproduce the typology of the Lorenz map—transition from a stable to a chaotic regime as the parameters (η, γ) are varied. The graphic confirms the correspondence between the two models, highlighting the general structure of phase transitions and instability zones.

Color map: Blue and green regions are periodic regimes, yellow–orange are aperiodic, and red are long-lived (metastable) aperiodic regimes. On the x-axis is the learning rate, and on the y-axis is the dissipation. The structures of the MRNN phase diagram and the Lorenz peak map in (*β*,) are shown in the first two diagrams, and a comparison is presented.

### Bifurcation Analysis

**Figure 6:**
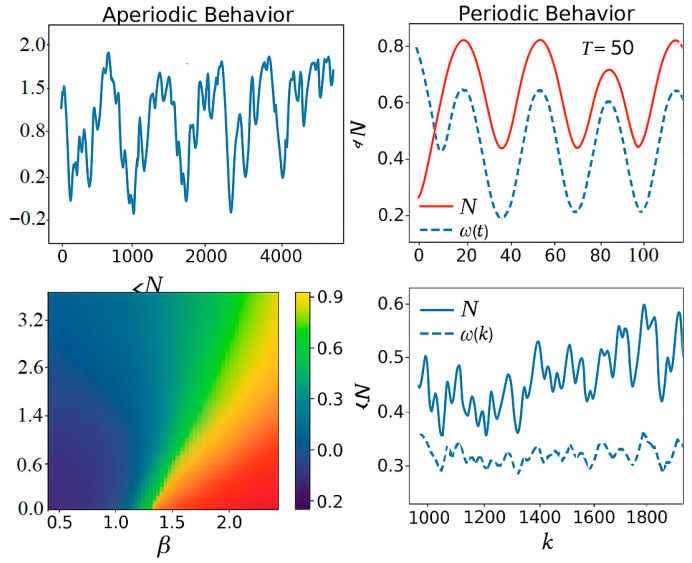
Comparative mapping of phase structures.

The Lorenz peak map for varying ρ∈ [20, 36] (integration with RK4, dt=0.01) is shown in Figure 6. The plot shows windows of periodicity and transition to chaos. MRNN v_s_. η for fixed γ = 0.25:

$$x_{t+1}=\left(1-\gamma \right)x_{t}+\eta W\;tanh{(x}_{t}),x_{t}\in R^{3},$$

where W is a fixed matrix with spectral radius 1 (dissipation by γ). We observe the peak values of x1(t) after transient burn-in. The diagram shows a fixed point → period-2 → chaos—the same hierarchy as in the peak map.

**Figure 7:**
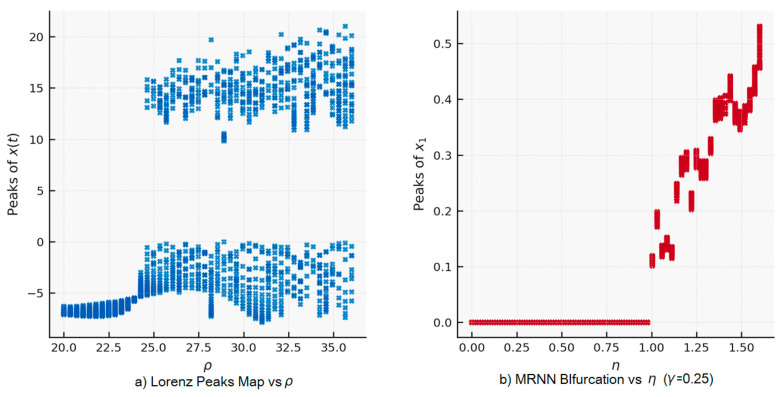
Lorenz ρ-sweep and MRNN η-sweep.

This diagram examines the behavior of the modified Kropotov–Pakhomov network (MRNN) when η is kept fixed and γ, the parameter of the dissipative feedback, is varied. The period map is a two-dimensional diagram that shows how the MRNN changes its behavior when we simultaneously vary η and γ (Figure 7). On the horizontal axis, η ∈ [0.2, 1.6]—the gain of the nonlinear term tanh(Wx). On the vertical axis: γ ∈ [0.0, 0.9]—the dissipative feedback. On the vertical axis are the peaks of the observed variable *x*_1_(*t*), extracted after eliminating transients. This means that for small γ and large η, there is a wide chaotic zone (denoted by 0), and dissipation is weak. The network behaves more “lively,” with larger amplitudes and a tendency towards multiperiodicity and chaos. At medium γ and η, distinguishable islands of periodicity (2, 3, 4,…,n) appear, analogous to the classical periodic structure in one-dimensional chaotic maps, and the network stabilizes. A zone is observed in which the amplitudes contract and the order “fixed point → period-2 → period-4 → chaos” is visible—the same classical bifurcation structure as in the Lorenz map. At large γ, the dissipation is strong. The system becomes practically single-periodic, or it contracts toward a fixed point. This diagram shows that adjusting the dissipativeness of γ plays an analogous role in changing the parameter ρ in the Lorenz peak map. This is precisely what supports the thesis that MRNN can reproduce the same classes of dynamical regimes as the Lorenz attractor.

Despite these positive results, the present work has several important limitations. First, the stability correspondence between the Lorenz peaks map and the MRNN is established under structural assumptions (existence of a one-dimensional invariant manifold and a smooth conjugacy) that are not proved in full generality. The validity of the reduction is supported by numerical evidence. The analysis is restricted to a specific MRNN architecture with a fixed dimension (64 neurons). Additionally, there is a specific Hebbian-type learning rule. Other architectures or activation functions may alter the fine structure of the attractor and its size. The discretization of the Lorenz system and the estimation of Lyapunov exponents introduce numerical errors, which may affect the quantitative boundaries of bifurcation regimes.

Future work will focus on researching these assumptions and strengthening the theoretical foundations of the model. A starting point is to develop rigorous criteria for the existence of invariant manifolds and conjugacies between discrete chaotic maps and neural recursions, possibly using normal form theory. A second direction is to extend the framework to other classes of chaotic flows and maps. A third direction is to integrate the proposed stability criterion into data-driven applications, such as adaptive control, cognitive modeling, and nonlinear signal processing, where MRNN-type networks could be used both as emulators and as tools for estimating local stability indices directly from time series.

## 5. Conclusions

This work presents stability analysis in discrete nonlinear systems by coupling the discrete Lorenz attractor with a modified Kropotov–Pakhomov neural network (MRNN). The one-dimensional peak map made it possible to create a compact but still accurate representation of the Lorenz system in discrete time, keeping its main bifurcation sequences and chaotic regimes. Formulating MRNN as a discrete nonlinear operator driven by the Bogdanov–Hebb learning rule allowed the adaptive extraction of stable subspaces and the identification of stability boundaries directly from the internal dynamics of the network. The principal contribution of the study is the established correlation between the peak-map multiplier and the spectral radius of the MRNN monodromy matrix, offering a universal criterion for asymptotic stability. Numerical experiments further confirmed that MRNN can reproduce stable, quasi-periodic, and chaotic behavior without external noise, establishing the network as an interpretable neural emulator of Lorenz-type discrete dynamics.

## Figures and Tables

**Table 1:** Optimization of parameters.

Modification	Effect on Dynamics	Interpretation
Remove constraint $h_{i}\geq 0$	Spontaneous activation; runaway excitation	Network becomes biologically implausible
Vary $A_{1,2},\;B_{1,2},C_{1,2}$ randomly by ±20%	Loss of periodic windows; chaotic bursts disappear	Efficiency dynamics become unstable
Remove condition (55)	Rapid changes in $W_{ij}^{0}$; loss of attractor compactness	The network no longer emulates Lorenz-like maps

## Data Availability

Data is contained within the article.

## Appendix A

### Appendix A.1. The Lorenz Time Discretization

The Lorenz time discretization algorithm is designed for arbitrary systems:

1. Initialization: Set X ← X_0_ Store X_0_ 2. For k = 0, 1, …, N − 1 do 3. Select integration scheme: X_(k+1)_ ← X_k_ + h ^x^ F(X_k_) K_1_ ← F(X_k) K_2_ ← F(X_k_ + 0.5 ^x^ h ^x^ K_1_) K_3_ ← F(X_k_ + 0.5 ^x^ h ^x^ K_2_) K_4_ ← F(_X_k_ + h ^x^ K_3_) X_(k+1)_ ← X_k_ + (h/6) ^x^ (K_1_ + 2 ^x^ K_2_ + 2 ^x^ K_3_ + K_4_) If || $X_{\left(k+1\right)}^{\left(n+1\right)}$− $X_{\left(k+1\right)}^{n}$|| ≤ tol then break End For X_(k+1)_ ← $X_{\left(k+1\right)}^{\left(n+1\right)}$ End Case 4. Store X_(k+1)_ 5. End For 6. Return {X_k_}

### Appendix A.2. Centroid Space-Time

Centroid space-time discretization algorithm—lattice

Input: {$u_{i}^{0}$}, $F_{h}$, h, *N*, tol, n Output: {$u_{i}^{0}$(k)} for k = 0, …, N 1: Apply boundary conditions to $u_{i}^{0}$ 2: for k = 0, 1, …, N − 1 do 3: if scheme == "Euler" then 4: for i = 1, …, M do 5: $u_{i}^{k+1}=u_{i}^{k}+hF_{h}(u_{i-1}^{k},u_{i}^{k},u_{i+1}^{k})$; 6: end for 7: else if scheme == "RK4" then 8: for i = 1, …, M do 9: K1, K2, K3, K4 10: end for 11: else if scheme == "Centroid" then 12: Initialize: $u_{i}^{k+1}$ ← $u_{i}^{k}$ for all i 13: for n = 0, 1, 2, …, n do 14: for i = 1, …, M do 15: $u_{i}^{n+1,\;\;k+1}$ ← $u_{i}^{k}$ 16: end for 17: Apply boundary conditions to {u_i^(n + 1, k + 1)} 18: if || $u_{i}^{n+1,\;\;k+1}$− $u_{i}^{n,\;\;k+1}$|| ≤ tol then 19: break 20: end if 21: end for 22: Set $u_{i}^{\;\;k+1}$ ← $u_{i}^{n+1,\;\;k+1}\;$ for all i 23: end if 24: Apply boundary conditions to {$u_{i}^{\;\;k+1}$)} 25: end for 26: return {$u_{i}^{\;\;k}$}, (k = 0, …, N)

We discretize the spatial domain on a uniform lattice and advance in time via either exlicit Euler/RK or an implicit centroid scheme. The implicit update solves, at each time layer *t*_k+1_, the fixed-point problem $u_{i}^{k+1}=u_{i}^{k}+F_{h}(\frac{u_{i-1}^{k}+u_{i-1}^{k+1}}{2},\frac{u_{i}^{k}+u_{i}^{k+1}}{2},\frac{u_{i+1}^{k}+u_{i+1}^{k+1}}{2})$, iterating until $\|u_{i}^{\left(n+1\right),k+1}-u_{i}^{\left(n\right),k+1}\|\leq tol;$

Boundary conditions are imposed after each iteration. The scheme extends verbtim to higher dimensions by replacing nearest-neighbor triples with the corresponding stecil N(i).

### Appendix A.3. Network Size Selection

Network Size Selection for the MRNN Lorenz Emulator

Algorithm Input: Target 1D map P (Lorenz peaks map), candidate sizes N ∈ {N_min, …, N_max}, tolerance ε_dyn for dynamical similarity. For each N in {N_min, …, N_max} do 1. Construct an N–neuron MRNN with fixed architecture and parameters constrained by (stability and boundedness). 2. Simulate the MRNN for the same range of control parameters as P (e.g., ρ for Lorenz and η for the MRNN). 3. Compute dynamical similarity metrics: (a) bifurcation pattern (number and order of period–doubling windows), (b) maximal Lyapunov exponent λ_max, (c) qualitative shape of invariant density. 4. Measure the mismatch Δ(N) between the MRNN and the Lorenz map in terms of the above metrics. End For Select N* = argmin_N Δ(N) subject to: (i) Δ(N*) ≤ ε_dyn (sufficient dynamical accuracy), (ii) the MRNN has a bounded attractor, (iii) sensitivity analysis remains interpretable (no excessive parameter redundancy). Output: Selected network size N* (in our case N* = 64).
